# Dual mRNA therapy restores metabolic function in long-term studies in mice with propionic acidemia

**DOI:** 10.1038/s41467-020-19156-3

**Published:** 2020-10-21

**Authors:** Lei Jiang, Ji-Sun Park, Ling Yin, Rodrigo Laureano, Eric Jacquinet, Jinsong Yang, Shi Liang, Andrea Frassetto, Jenny Zhuo, Xinhua Yan, Xuling Zhu, Steven Fortucci, Kara Hoar, Cosmin Mihai, Christopher Tunkey, Vlad Presnyak, Kerry E. Benenato, Christine M. Lukacs, Paolo G. V. Martini, Lin T. Guey

**Affiliations:** Moderna Inc., 200 Technology Square, Cambridge, MA 02139 USA

**Keywords:** Drug safety, Pharmacology, Metabolic disorders

## Abstract

Propionic acidemia/aciduria (PA) is an ultra-rare, life-threatening, inherited metabolic disorder caused by deficiency of the mitochondrial enzyme, propionyl-CoA carboxylase (PCC) composed of six alpha (PCCA) and six beta (PCCB) subunits. We herein report an enzyme replacement approach to treat PA using a combination of two messenger RNAs (mRNAs) (dual mRNAs) encoding both human PCCA (hPCCA) and PCCB (hPCCB) encapsulated in biodegradable lipid nanoparticles (LNPs) to produce functional PCC enzyme in liver. In patient fibroblasts, dual mRNAs encoded proteins localize in mitochondria and produce higher PCC enzyme activity vs. single (PCCA or PCCB) mRNA alone. In a hypomorphic murine model of PA, dual mRNAs normalize ammonia similarly to carglumic acid, a drug approved in Europe for the treatment of hyperammonemia due to PA. Dual mRNAs additionally restore functional PCC enzyme in liver and thus reduce primary disease-associated toxins in a dose-dependent manner in long-term 3- and 6-month repeat-dose studies in PA mice. Dual mRNAs are well-tolerated in these studies with no adverse findings. These studies demonstrate the potential of mRNA technology to chronically administer multiple mRNAs to produce large complex enzymes, with applicability to other genetic disorders.

## Abstract (web summary)

Propionic acidemia is a serious pediatric inherited disorder with no effective treatments. Here the authors demonstrate that delivering dual mRNAs as an enzyme replacement approach can be used as an effective therapy in a mouse model of propionic acidemia, with potential applicability to chronically administer multiple mRNAs in other genetic disorders.

## Introduction

Recent advances in mRNA technology have transformed the prospect of systemic mRNA encapsulated in LNPs to produce therapeutic intracellular^1–3^ and secreted proteins^4–7^ using the cell’s endogenous translational machinery as a potential enzyme-replacement approach. Key advantages of mRNA technology include its transient, non-integrating nature, and the ability to deliver multiple mRNAs to encode complex multimeric proteins^8,9^. However, the long-term efficacy and safety of exogenous mRNA remain to be comprehensively evaluated in preclinical studies.

PA is a devastating pediatric disorder caused by PCC enzyme deficiency, which results in impaired propionate metabolism and abnormal accumulation of toxic metabolites, including primary disease markers 2-methylcitrate (2MC), 3-hydroxypropionate (3HP), and propionylcarnitine (C3)^10–16^. Inhibition of ureagenesis by these metabolites and propionyl CoA (the substrate for PCC) also leads to hyperammonemia^10,12^. PCC is a large 750-kDa heterododecamer enzyme^17^. The alpha (PCCA) and beta (PCCB) subunits of the enzyme are 72 and 54 kDa, respectively^18^, and thus require relatively large externally manufactured mRNA constructs (>1.5 kb each) to encode these subunits in full length (Supplementary Table 1). Two disease subtypes exist: PCCA-deficient (Type I) and PCCB-deficient (Type II). PCCB is intrinsically unstable if not in complex with PCCA^12,19^, and thus PCCA-deficient patients are typically deficient in both protein subunits. PCCB-deficient patients can have lower levels of PCCA protein as well^12,19,20^.

Herein, we report an enzyme-replacement approach to address both PA disease subtypes using dual mRNAs encoding both hPCCA and hPCCB subunits. Dual mRNAs encode functional PCC enzyme with proper mitochondrial localization, and thus restore metabolic function in mouse liver with PA. Preclinical experiments that include 3- and 6-month studies in a relevant murine disease model demonstrate the long-term therapeutic potential of a combination mRNA therapy approach for a rare metabolic disorder.

## Results

### mRNA-encoded functional PCC enzyme localized in the mitochondria

**Fig. 1:**
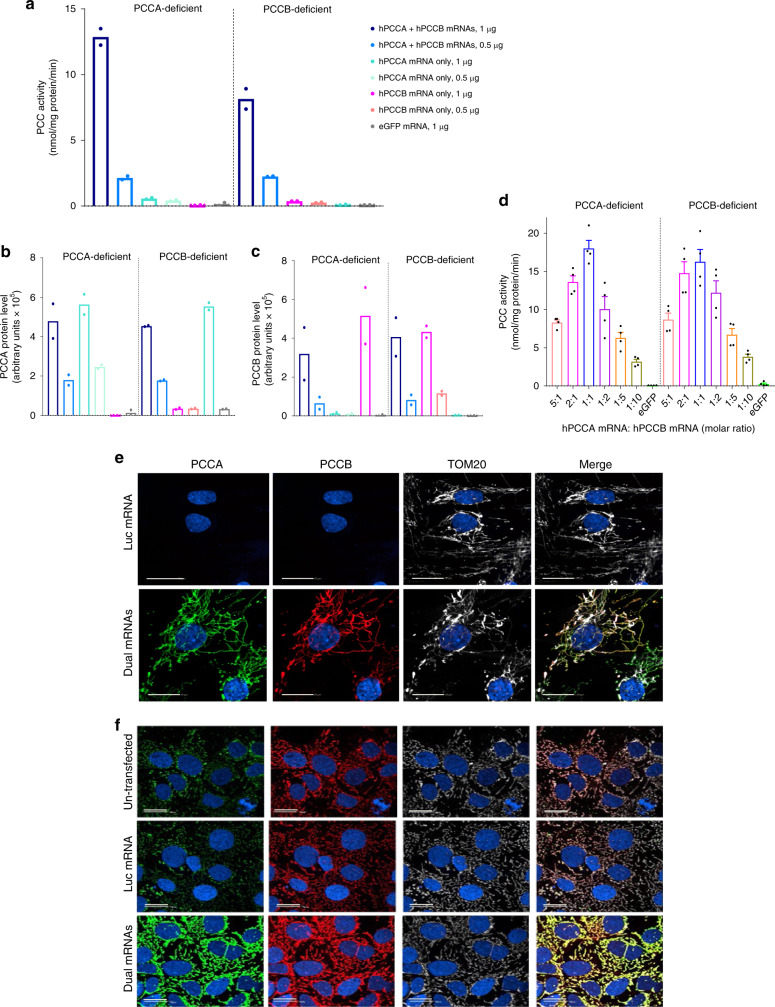
Dual mRNAs encode functional PCC with proper mitochondrial localization in patient-derived fibroblasts and human Hep3B cells. **a**–**c** Comparison of co-transfection of hPCCA+hPCCB (dual) mRNAs with single transfection of hPCCA or hPCCB mRNA alone. Human fibroblasts isolated from a PCCA-deficient PA patient and a PCCB-deficient PA patient (*n* = 2 per condition) were transfected with 0.5 or 1 µg of the total dual mRNAs (1:1 molar ratio), hPCCA mRNA alone, or hPCCB mRNA alone, or 1 μg of eGFP control mRNA for 24 h. Cells were lysed, and mitochondrial matrix fractions were collected to assess PCC enzymatic activity by a radiometric assay (**a**), and protein levels of PCCA (**b**) and PCCB (**c**) by capillary electrophoresis. **d** Identification of the optimal molar ratio of hPCCA mRNA:hPCCB mRNA. Patient fibroblasts were transfected with 0.67 nM (1 µg) of total dual mRNAs across a range of molar ratios of hPCCA mRNA:hPCCB mRNA or with the same molar concentration of eGFP mRNA (*n* = 4 per condition) for 24 h. PCC activity in the cellular mitochondrial matrix fractions was measured. Data are shown as mean ± SEM. **e**, **f** Subcellular localization of dual mRNAs-encoded PCC subunits in PCCA-deficient patient fibroblasts and human liver-derived Hep3B cells. Cells were transfected with 25 ng of dual mRNAs or luciferase (Luc) control mRNA for 24 h, and were stained for PCCA, PCCB, and TOM20, a mitochondrial marker. Scale bars are 20 µm. Representative images are shown from *n* = 24 replicates.

Transfection of dual mRNAs in patient fibroblasts obtained from both PA subtypes resulted in dose-dependent increases in PCC enzyme activity and protein subunits (Fig. 1a–c and Supplementary Fig. 1a–c). PCC enzyme activity was 5–24× greater in fibroblasts transfected with dual mRNAs vs. hPCCA or hPCCB mRNA alone (Fig. 1a), suggesting that both mRNA-encoded PCC protein subunits contribute to increasing PCC activity. The optimal molar ratio of hPCCA mRNA:hPCCB mRNA yielding the highest PCC enzyme activity in fibroblasts was 1:1 (Fig. 1d). PCC activity increased similarly in PCCA- and PCCB-deficient fibroblasts, suggesting that dual mRNA therapy could be effective for both subtypes. Mitochondrial localization of mRNA-encoded proteins was confirmed by immunofluorescence analysis of human fibroblasts (Fig. 1e and Supplementary Fig. 1d, e) and liver-derived Hep3B cells (Fig. 1f).

### Kinetics of mRNA-encoded PCC subunits and activity in the liver

**Fig. 2:**
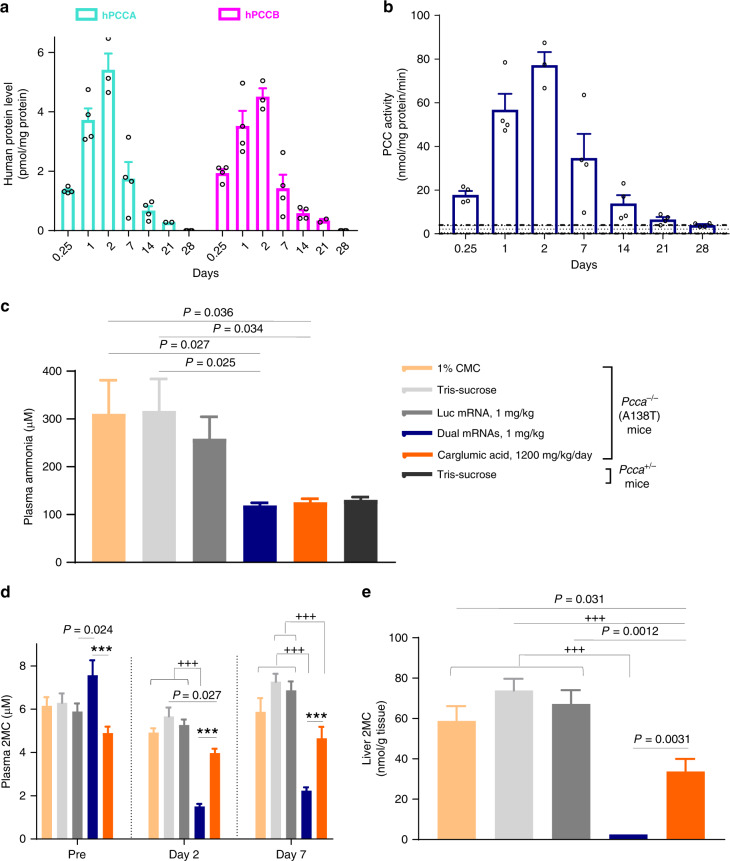
Kinetics of dual mRNAs-encoded hepatic PCC and comparison of dual mRNA therapy with carglumic acid in PA mice. **a**, **b** Kinetics of dual mRNAs-encoded hepatic PCC subunits and enzyme activity. PA hypomorphic mice of mixed gender were administered a single IV bolus injection (1 mg/kg) of dual mRNAs (*n* = 4 mice/time point) or Luc control mRNA (*n* = 3 mice/time point) encapsulated in LNPs and were sacrificed at various time points. **a** Absolute quantification of hPCCA and hPCCB proteins in livers from dual mRNAs-injected PA mice by LC-MS/MS using signature peptides for wild-type (WT) hPCCA and hPCCB protein sequences. hPCCA and hPCCB were below the LLOQ in all livers from Luc control mRNA-injected mice. **b** PCC enzyme activity in hepatic mitochondria was measured. Shaded bars represent the range of endogenous PCC enzyme activity levels from all Luc mRNA-injected mice across all time points (*n* = 21). **c**–**e** Substantial reductions in plasma ammonia and primary disease biomarkers due to dual mRNAs in contrast to carglumic acid. PA hypomorphic female mice were administered a single IV bolus dose of Tris-sucrose buffer (*n* = 11) or 1 mg/kg of dual mRNAs or Luc control mRNA (*n* = 12) encapsulated in LNPs and were sacrificed 7 days post injection. Additional PA hypomorphic female mice were administered repeat doses of 1% carboxymethyl cellulose (CMC, *n* = 11) or carglumic acid (1200 mg/kg/day, twice daily, *n* = 12) via oral gavage for 7 consecutive days and then sacrificed. Unaffected *Pcca*^*+/−*^ mice (*n* = 9) were IV injected with Tris-sucrose buffer as control. Mice were bled at study days 0 (pre-treatment), 2, and 7. Plasma ammonia (**c**) and liver 2MC (**e**) at day 7 upon sacrifice, and plasma 2MC (**d**) were assessed. Data are shown as mean ± SEM. Plasma and liver 2MC levels in unaffected mice were <LLOQ. *P* values were obtained from Tukey’s post hoc pairwise multiple comparison test following a one-way or two-way ANOVA, and are provided in the source data. ***P < 0.001 comparing dual mRNA therapy with carglumic acid. ^+++^*P* < 0.001 comparing dual mRNAs or carglumic acid group with control groups (1% CMC, Tris-sucrose, and Luc mRNA). Pre, pre-treatment on day 0.

A single intravenous (IV) bolus dose of dual mRNAs formulated in biodegradable LNPs (1 mg/kg) in a hypomorphic murine disease model (*Pcca*^*−/−*^[p.A138T]) resulted in therapeutically relevant protein levels of hPCCA and hPCCB, with C_max_ values of 5.4 and 4.5 pmol/mg total liver protein, respectively (Fig. 2a), which are similar to endogenous protein levels in human liver (4.5 and 3.8 pmol/mg for PCCA and PCCB, respectively). Equimolar production of hepatic hPCCA and hPCCB was observed with AUC_(0-inf)_ values of 927 and 833 h *pmol/mg protein, respectively. The kinetics of mRNA-encoded PCC protein subunits and enzyme activity in the liver showed a substantial increase at 6 h, peaked at 2 days (*T*_max_), and remained detectable at 21 days post-dose (hepatic *T*_1/2_ 5.3–6.7 days, Fig. 2a, b). These data indicate that dual mRNAs could potentially be used to rapidly restore functional enzyme in the liver during life-threatening acute metabolic decompensations, a hallmark of PA.

### Treatment with dual mRNAs in contrast to carglumic acid

The pharmacodynamic (PD) effects of dual mRNAs were compared to carglumic acid (Carbaglu^®^), a ureagenesis-activating agent approved in the European Union for the treatment of hyperammonemia due to PA^11,21,22^, in a 1-week study in PA mice. Plasma ammonia, a potent neurotoxin and clinically meaningful endpoint, was normalized in mice administered dual mRNAs (single IV dose, 1 mg/kg) and carglumic acid (oral gavage twice daily, 1200 mg/kg/day) (Fig. 2c). In contrast to carglumic acid, dual mRNAs additionally led to pronounced reductions in plasma primary biomarkers (2MC, 3HP, and C3 normalized to acetylcarnitine C2 [C3/C2 ratio]) and tissue (liver, heart, and kidney) 2MC, and increased PCC activity in liver (Fig. 2d, e and Supplementary Fig. 2a–g). Therefore, unlike carglumic acid, dual mRNAs restored the physiologic process of propionate metabolism in the liver in PA mice, thus addressing the underlying metabolic defect.

### Sustained pharmacology in long-term repeat-dose studies

**Fig. 3:**
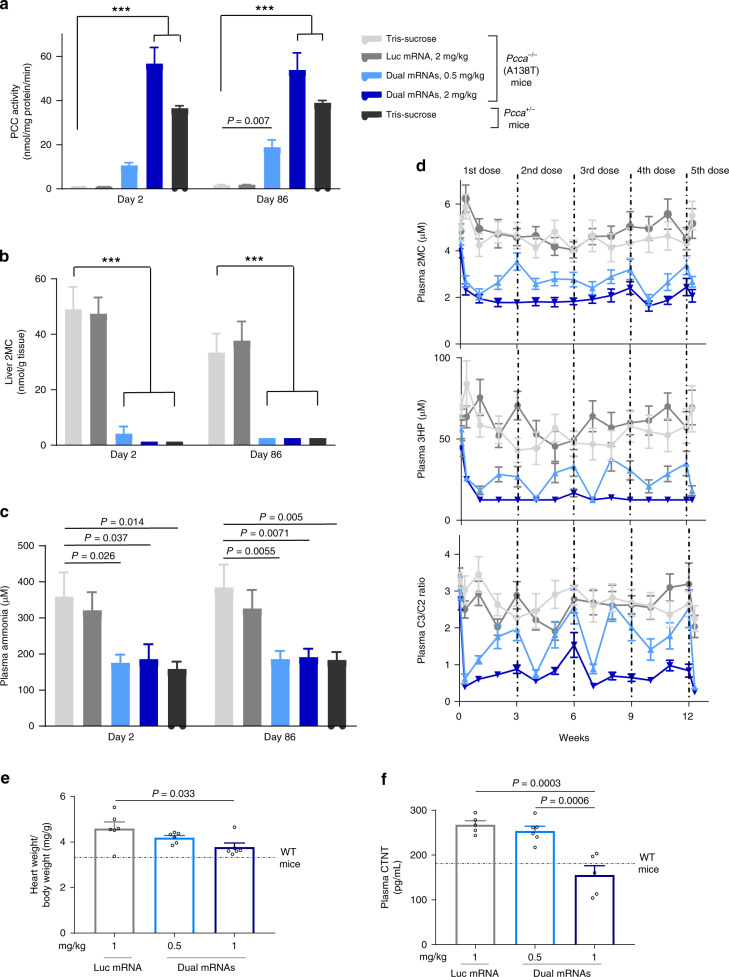
Long-term efficacy in 3- and 6-month repeat-dose studies in PA hypomorphic mice. **a**–**d** 3-month study showed sustained and reproducible biomarker reductions due to dual mRNAs. PA hypomorphic mice of mixed gender were IV bolus administered 0.5 or 2 mg/kg dual mRNAs or 2 mg/kg Luc control mRNA or Tris-sucrose buffer (*n* = 12/group/sacrifice time point) every 3 weeks for 12 weeks. Mice were sacrificed 2 days after the first dose (day 2) and 2 days after the last dose (day 86). PCC enzyme activity in liver mitochondria (**a**), liver 2MC (**b**), and plasma ammonia (female mice only as male PA mice do not exhibit elevated ammonia levels) (**c**) were assessed on days 2 and 86. **d** Plasma primary disease biomarkers were assessed weekly throughout the 3-month study. Biomarker levels in unaffected heterozygote mice (*n* = 24) were <LLOQ for plasma 2MC and 3HP, and <0.13 for plasma C3/C2 ratio at all time points. **e**, **f** Amelioration of heart weight/body weight ratio and plasma CTNT levels in the 6-month study. PA hypomorphic female mice received a total of six IV bolus doses of 0.5 or 1 mg/kg dual mRNAs or 1 mg/kg Luc control mRNA (*n* = 6/group) at weeks 0, 7, 11, 15, 20, and 24. Mice were sacrificed 2 days after the last dose. Age-matched untreated female WT mice (*n* = 6) were added to the end of the study at sacrifice. Hearts were weighed and normalized to body weights (**e**), and plasma CTNT was assessed (**f**). The dotted lines indicate the mean values obtained from *n* = 6 untreated WT age-matched female mice. Levels of heart weight/body weight and plasma CTNT in these WT mice were 3.3 ± 0.13 mg/g and 181.1 ± 43.9 pg/mL, respectively. Data are presented as mean ± SEM. *P* values were obtained from Tukey’s or Dunnett’s post hoc pairwise multiple comparison test following a one-way or two-way ANOVA, and are provided in the source data. ***P < 0.001.

Due to the transient nature of mRNA therapy, an important consideration for an mRNA-based enzyme-replacement approach is whether exogenous mRNA can be safely administered with consistent pharmacology upon chronic repeat dosing. To address these outstanding questions, we conducted two long-term studies lasting 3- and 6-months in PA hypomorphic mice. In the 3-month study, 0.5 and 2 mg/kg of dual mRNAs encapsulated in LNPs were IV administered every 3 weeks (dose levels and regimen determined from a single-administration dose-range-finding study shown in Supplementary Fig. 3). Dual mRNAs treatment led to dose-dependent increases in hepatic PCC activity and equimolar production of PCC protein subunits 2 days following the first and last dose (Fig. 3a and Supplementary Fig. 4a). As a direct consequence of restoring PCC activity in the liver, hepatic 2MC was normalized in dual mRNAs-treated mice (Fig. 3b). Furthermore, plasma ammonia was normalized after single and repeat administration of dual mRNAs (Fig. 3c).

Dose-dependent reductions in plasma primary biomarkers (2MC, 3HP, C3/C2 ratio) were observed throughout the 3-month study (Fig. 3d and Supplementary Fig. 4b, c). Plasma biomarker reductions were reproducible and sustained for weeks following each dose administration with no attenuation of PD response, demonstrating the long-term bioactivity of dual mRNAs. This long-term pharmacology finding was reproduced in the 6-month study, in which PA mice were IV administered dual mRNAs (0.5 or 1 mg/kg) at approximately monthly dosing intervals (Supplementary Fig. 5a). Similar to the 3-month study, hepatic PCC enzyme activity was increased in a dose-dependent manner due to dual mRNAs treatment at the end of the 6-month study (Supplementary Fig. 5b). These collective data suggest that a small amount of restored enzyme is needed to elicit a PD response; indeed, patients with residual PCC enzyme activity typically have a milder disease course than patients with no PCC enzyme activity^23^.

Cardiac abnormalities such as cardiomyopathy and cardiac rhythm abnormalities are common clinical findings in patients with PA that can be life-threatening^11,13,16,24^. Notably, liver transplantation is a potential treatment option to increase PCC enzyme in severely affected patients and has been reported to reverse cardiomyopathy in several case reports^25–28^. At the end of the 6-month study, attenuation of elevated heart weight/body weight ratio and plasma cardiac troponin T (CTNT), a myocardial injury marker^29^, was observed in dual mRNAs-treated PA hypomorphic mice (Fig. 3e, f). Since dual mRNAs did not increase PCC activity in the heart, these cardiac improvements (i.e., prevention of cardiac abnormalities) were likely due to decreased levels of circulating toxic metabolites. However, brain natriuretic peptide (BNP), a marker of cardiac dysfunction, was not elevated in heart tissue mRNA, as previously described^30^. No cardiac abnormalities were observed between PA and unaffected mice in the 3-month study (likely due to age). Furthermore, all studies were initiated in mice at 4–5 months of age, prior to the development of cardiomyopathy (and thus none of the mice had pre-existing cardiomyopathy). Thus, these cardiac findings warrant further investigation in additional preclinical studies and upcoming clinical trials.

### Long-term repeat dosing of dual mRNAs is well-tolerated

**Fig. 4:**
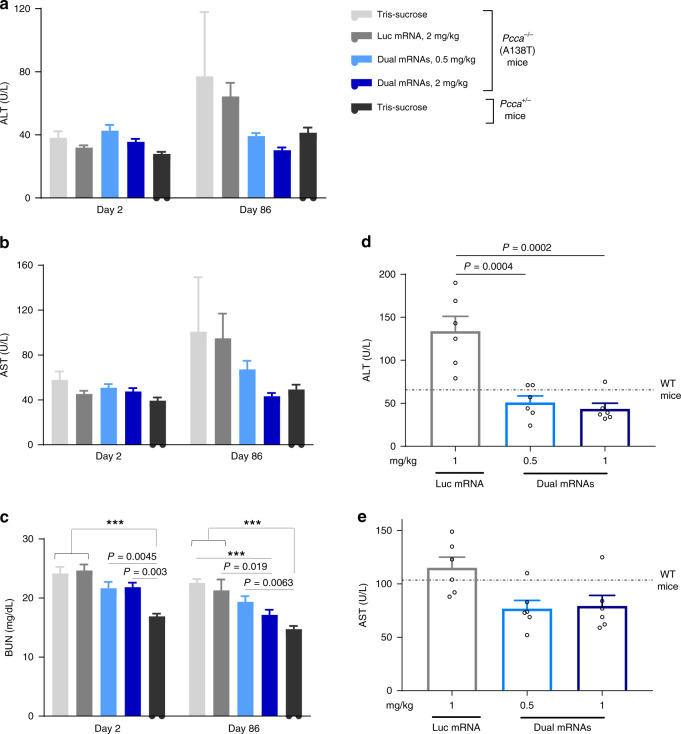
Dual mRNAs are well-tolerated in 3- and 6-month repeat-dose studies in PA hypomorphic mice. **a**–**c** Select clinical chemistry parameters (serum ALT, AST, and BUN) in PA hypomorphic and unaffected mice (*n* = 12/group/sacrifice time point) from the 3-month study. **d**, **e** Select clinical chemistry parameters (plasma ALT and AST) in PA hypomorphic female mice (*n* = 6/group) from the 6-month study. The dotted lines indicate the mean values from *n* = 5 untreated WT age-matched female mice. Levels of plasma ALT and AST in these untreated WT mice were 65.60 ± 9.49 and 103.60 ± 14.15 U/L, respectively (one outlier was excluded). Data are presented as mean ± SEM. *P* values were obtained from Tukey’s post hoc pairwise multiple comparison test following a one-way or two-way ANOVA, and are provided in the source data. ***P < 0.001. ALT alanine aminotransferase, AST aspartate aminotransferase.

Long-term administration of dual mRNAs encapsulated in LNPs was well-tolerated with no adverse reactions in 3- and 6-month studies in PA mice. Throughout both studies, there were no deleterious dual mRNAs-related clinical observations and body weight effects (Supplementary Fig. 4d and Fig. 5c). Of note, body weight was evaluated as a safety parameter, as there were no differences in body weights of PA vs. unaffected mice (in contrast to a previous report^30^). Throughout the duration of both long-term studies, body weights were similar between all study groups, including unaffected control mice. Additionally, no toxicologically related findings across all clinical chemistry parameters were observed at the end of both studies (Fig. 4, Supplementary Data 1, and Supplementary Table 2). Liver transaminases and blood urea nitrogen (BUN) trended toward normal unaffected levels in dual mRNAs-treated mice. A prior limitation of polyethylene glycolated (PEGylated) lipid-delivery systems has been the induction of accelerated blood clearance of the LNPs partly due to the formation of antibodies against the PEGylated-surface lipids^31^. In contrast, here we observed no increase in anti-PEG IgM at the end of the 3-month study (Supplementary Fig. 4e). Histopathology examination across a full panel of tissues (Supplementary Table 3) in the 3-month study revealed no dual mRNAs-related macroscopic findings and limited microscopic findings in the liver (Supplementary Fig. 4f). Specifically, minimal-to-mild increased mitosis was observed following a single dose of dual mRNAs at 2 mg/kg. At the end of the 3-month study, LNP-related microscopic changes in the liver consisted of increased incidence of minimal-to-mild perivascular mixed cells infiltrate (Supplementary Table 4) characterized by the accumulation of macrophages and lymphocytes admixed with a few neutrophils, which was mainly observed near the centrilobular vein. The overall safety and tolerability profile observed in these long-term studies are consistent with previous systemic mRNA safety evaluations in mice, rats, rabbits, and non-human primates using a similar biodegradable lipid-delivery system^2,5,32^.

## Discussion

The studies reported herein provide a comprehensive preclinical evaluation of the long-term efficacy and safety of a combination of mRNA therapy, with applicability to other genetic disorders. Significant decreases in plasma primary biomarkers due to treatment of dual mRNAs were consistently observed in short- and long-term studies conducted in PA hypomorphic mice. However, these primary disease biomarkers were not normalized to levels observed in unaffected mice. This is consistent with the liver transplantation experience in patients with PA, which show a reduction, but not full normalization, of metabolite profiles post transplant^27,28,33^ due to the accumulation of disease-associated metabolites in extrahepatic tissues, such as skeletal muscle. Although liver transplantation does not completely normalize disease-associated metabolites, it has been shown to improve clinical outcomes, specifically significantly reducing the occurrence of life-threatening metabolic decompensations, improving the overall quality of life, and improving cardiac function in patients with PA^27,28,33^.

In the current studies in PA hypomorphic mice, dual mRNAs increased PCC enzyme activity in the liver and led to significant reductions in disease-associated metabolites, including ammonia. Due to the lack of available reagents suitable to detect mRNA-encoded proteins in PA mouse livers, we were unable to perform immunohistochemistry in the PA mouse studies. However, our previous studies with the same LNP delivery vehicle have shown a broad expression of mRNA-encoded protein in mouse liver^2^. Additionally, the function of the PCC enzyme is to catalyze the carboxylation of propionyl-CoA to methylmalonyl-CoA, which occurs in the mitochondria^12^; thus, the substantial decreases in disease-associated metabolites (all of which are by-products of propionyl-CoA accumulation) indicate the mRNA-encoded PCC enzyme complex was properly localized in the mitochondria. The in vivo pharmacology data collectively indicate that dual mRNAs restored propionate metabolism in these mice. In vivo propionate oxidation has been shown to be a promising, non-invasive, and sensitive approach to assess the overall level of propionate metabolism^34^. Although the current studies did not evaluate in vivo propionate oxidation in PA mice, this readout could be considered in upcoming clinical trials.

Whether the mice treated with dual mRNAs developed anti-PCCA and/or anti-PCCB antibodies was not evaluated. As the PCC subunits are expressed intracellularly and trafficked to the mitochondria, it is unlikely that an antibody response against the mRNA-encoded proteins would be induced. Furthermore, if anti-PCCA and/or anti-PCCB antibodies develop, they would unlikely neutralize the intracellular mitochondrial enzyme complex and thus will not affect enzyme activity. This is supported by the consistent pharmacologic response after each dose of dual mRNAs in both long-term studies, suggesting that even if antibodies were produced against the LNP or mRNA-encoded proteins, there was no neutralizing effect. Nevertheless, anti-PCCA and anti-PCCB antibodies will be assessed in the upcoming Phase 1/2 trial (Clinicaltrials.gov identifier NCT04159103) in patients with PA receiving dual mRNAs (i.e., mRNA-3927).

Previously published preclinical gene therapy reports with adeno-associated virus 2/8 (AAV8)-based delivery of PCCA cDNA showed durable amelioration of disease-associated metabolites lasting for 1.5 years in adult PA hypomorphic mice^35^. We did not include an AAV8-PCCA cDNA-positive control in the current set of studies, as the intent of these studies was to support a first-in-human (FIH) clinical trial evaluating dual mRNAs. However, the published gene therapy approach focuses on replacing a single subunit of the deficient heterododecamer enzyme complex, and thus addresses only a subset of PA patients. In contrast, the dual mRNAs approach delivers two mRNAs encoding both enzyme subunits, and thus, is intended to treat all patients with PA (both subtypes). Additionally, one of the limitations of gene therapy is delayed therapeutic onset^36^. Upon administration of AAV8-hPCCA in the same PA murine model, PCCA protein levels were not significantly increased in the liver until 10 days post injection^35^. In contrast, a rapid onset was observed for dual mRNAs, with significant increases of hepatic PCC protein subunits and enzyme observed as early as 6 h post-dose (Fig. 2a). The therapeutic onset of potential novel genomics medicines may be particularly important during life-threatening acute metabolic decompensations. Another limitation of AAV gene therapy is its limited applicability to patients under ~12-years old due to their ongoing organ growth^37^, as opposed to mRNA therapy, which is a viable option for infants, which constitutes the majority of the patient population of PA.

*Pcca*^*−/−*^ (A138T) hypomorphic mice do not exhibit other clinical manifestations observed in PA patients, such as metabolic decompensations, neurologic manifestations, or significant growth retardation. Thus, the evaluation of clinically meaningful endpoints in *Pcca*^*−/−*^ (A138T) mice was limited to hyperammonemia and cardiac parameters. In contrast to Guenzel et al.^30^, we did not observe significant mortality in this mouse model. Specifically, we have observed a mortality rate of ~2% in the PA hypomorphic mouse colony. In the current set of studies, we observed a mortality rate of 2.4% (6/250) in PA hypomorphic mice, with deaths randomly distributed across study groups. Due to the low mortality rate observed in PA hypomorphic mice, these studies were not powered to detect differences in survival. Similarly, compared to previous publications^38,39^, we observed a milder mitochondrial dysfunction in PA hypomorphic mice (Supplementary Fig. 6). Only small alterations (<1.5-fold) in levels of some oxidative protein markers in the liver and heart were observed (Supplementary Table 5 and Supplementary Fig. 6a–c). In addition, mitochondrial genomes in the liver, heart, and brain of *Pcca*^*−/−*^ (A138T) mice were not significantly different from WT mice as indicated by copy numbers and/or aggregate mutation rates of mitochondrial DNAs (Supplementary Fig. 6d, e). Severe PA mouse models do exist, such as *Pcca*^*−/−*^ and *Pccb*^*−/−*^ global knockout mice. However, these mice are neonatal lethal and die within 36 h of birth^30^, making it difficult to evaluate the potential of an IV administered product that requires repeat dosing.

Altogether, these data provide compelling preclinical proof-of-concept for dual mRNA therapy as a potential disease-modifying treatment that addresses the underlying metabolic defect for PA (PCCA- and PCCB-deficient). These studies comprehensively establish the long-term efficacy and safety for a systemic combination mRNA therapeutic as an enzyme-replacement approach for a previously undruggable target, and one that could be applied to other genetic disorders. An outstanding question for systemic mRNA therapy is the ability to chronically administer repeat doses; our long-term (3- and 6-month) data show reproducible and consistent efficacy after each dose with a favorable safety profile. Furthermore, in a head-to-head preclinical study with an ammonia-lowering agent that is approved in the European Union, dual mRNAs addressed the underlying metabolic defect in contrast to carglumic acid. The studies described herein support the clinical development of dual mRNA therapy for this devastating pediatric disorder with no effective treatment options other than liver transplantation.

## Methods

### mRNA production and formulation

Codon-optimized mRNAs encoding hPCCA and hPCCB proteins were synthesized in vitro by T7 RNA polymerase-mediated transcription^1^. Each mRNA was initiated with a cap, followed by a 5′ untranslated region (UTR), an open-reading frame (ORF) encoding hPCCA or hPCCB, a 3′ UTR and a polyadenylated tail. Different codon-optimized ORFs in the hPCCA and hPCCB constructs (Supplementary Table 1) encoded each protein. Uridine was globally replaced with 1-methylpseudouridine. All experiments evaluating dual mRNAs utilized hPCCA and hPCCB mRNAs in a fixed 1:1 molar ratio, with the exception of the in vitro experiment to determine the optimal ratio of mRNAs (Fig. 1d).

For in vivo IV delivery, LNP formulations were generated^1^. Briefly, mRNA was mixed with lipids at a molar ratio of 3:1 (mRNA:lipid) and exchanged into the final storage buffer using tangential flow filtration. Formulated LNPs had particle sizes of 80–100 nm, >80% encapsulation by RiboGreen, and <10 EU/mL endotoxin levels. Formulations encapsulating one or two mRNA(s) were similar with respect to particle size and encapsulation efficiency.

### In vitro mRNA transfection in human fibroblasts

Human fibroblasts, isolated from a PCCA-deficient PA patient (#GM371 cell line with *PCCA* mutations c.1788G>A [p.W596X] and c.1561-1566delinTATTGCCAATAACC) and a PCCB-deficient PA patient (#GM1298 cell line with *PCCB* mutations c.1218_1231delinsTAGAGCACAGGA and c.1606A>G [p.N536D]), were purchased from Coriell. Patient fibroblasts (3–4 × 10^5^ cells/well in six-well plates) were transfected with 0.5 or 1 μg of total hPCCA+hPCCB (dual) mRNAs, or hPCCA or hPCCB mRNA alone, or 1 μg of eGFP control mRNA for 24 h using lipofectamine MessengerMAX (Thermo Fisher Scientific). Cells were harvested and lysed in mitochondrial lysis buffer containing 0.5% Triton X-100, 1 mM DTT, and 10 mM HEPES, pH 7.4 supplemented with protease inhibitors (Sigma). Cell lysates were further processed by six freeze/thaw cycles and centrifuged at 18,000 × *g* for 15 min to obtain the mitochondrial matrix in the supernatant for further analysis. To determine if dual mRNAs-encoded PCC subunits have the proper subcellular localization in the mitochondria, human fibroblasts or Hep3B cells (9000 cells/well plated in a 96-well plate) were transfected with 25 ng of dual mRNAs or luciferase (Luc) control mRNA for 24 h with lipofectamine MessengerMAX. Transfected cells were fixed in 4% paraformaldehyde and stained with an anti-PCCA mouse monoclonal antibody (Santa Cruz #sc-393527, 1:100), an anti-PCCB rabbit polyclonal antibody (Novus #NBP1-85886, 1:1000), and an anti-TOM20 mitochondrial marker antibody (Abcam #ab205486, 1:1000). Samples were imaged on an Opera Phenix spinning disk confocal microscope (PerkinElmer), using a ×63 water immersion objective (NA 1.4). Sixteen fields of view (~40 cells each) were imaged for each sample. The TOM20 mitochondrial marker was imaged with the 488-nm laser line, PCCA was imaged with the 647-nm laser line, and PCCB was imaged with the 561-nm laser line. The nuclear stain was imaged with the 405-nm laser line. A z-stack of five optical sections spanning 2 µm were acquired for all three channels.

### Hypomorphic mouse model of PA

The mouse model of PA utilized in the current studies was *Pcca*^*−/−*^ (A138T) mice, wherein the lethal *Pcca*^*−/−*^ phenotype is rescued by a transgene bearing an p.A138T mutant of the human PCCA protein (p.A138T)^30^. The knockout mice homozygous for p.A138T on a FVB background strain, survive to adulthood but having only ~2% of wild-type (WT) hepatic PCC activity, recapitulate metabolic characteristics, and some clinical phenotypes observed in PA patients. Specifically, these mice exhibit elevations of disease-associated metabolites and mild cardiomyopathy when hearts from 8-month-old mice were examined^30^. Experimental protocols were approved by the Institutional Animal Care and Use Committee at Moderna and complied with all relevant ethical regulations regarding the use of research animals. Mice were housed under the following conditions: temperature—68 °F to 79 °F (20–26 °C), humidity—30–70%, dark/light cycle—an automatically controlled 12-h light:12-h dark–light cycle was maintained. *Pcca*^*−/−*^ (A138T) mice received IV bolus injection(s) of dual mRNAs or Luc control mRNA formulated in LNPs or Tris-sucrose control buffer via the tail vein. Carglumic acid (Sigma) was dissolved in 1% aqueous carboxymethyl cellulose (CMC), and either this suspension or 1% CMC alone as a buffer control was administered via oral gavage. Additional cohorts of unaffected *Pcca*^*+/−*^ heterozygote or WT mice were included in studies as controls. An equal proportion of male and female mice were used in all studies to avoid gender biases, with the exception of the 1-week carglumic acid study and the 6-month chronic study. The 1-week carglumic acid study was limited to female mice, as only female PA mice exhibit hyperammonemia (i.e., male PA mice do not exhibit elevated plasma ammonia levels). The 6-month study, similarly, was conducted in female PA mice, as they have elevated metabolite concentrations compared to male mice consistent with the literature^35^. The reason for this gender discrepancy is unclear, and this gender discrepancy in biochemical parameters has not been described in patients. Mice were 4–5 months old at the initiation of all in vivo studies.

### Mitochondrial isolation and protein quantification

At sacrifice, livers were harvested and homogenized in a homogenization buffer containing 10 mM Trizma base-MOPS, 1 mM EGTA-Trizma base, and 200 mM sucrose supplemented with protease inhibitor. The liver homogenate was further processed to isolate the mitochondria after two steps of centrifugation, at 600×*g* for 10 min and 7000*×g* for 20 min sequentially. The resultant mitochondrial pellet was resuspended in mitochondrial lysis buffer. After six freeze/thaw cycles, the fraction was centrifuged at 18,000×*g* for 15 min to obtain the mitochondrial matrix. Protein estimation was performed using the bicinchoninic acid method (BCA protein assay kit; Thermo Scientific Pierce) according to the manufacturer’s recommendations. Semiquantitative analysis of PCCA and PCCB protein expression was achieved by capillary electrophoresis (Wes, ProteinSimple) based on the manufacturer’s recommendations. For detection of PCCA and PCCB, a polyclonal anti-PCCA antibody (Proteintech #21988-1-AP, 1:1000) and a polyclonal anti-PCCB antibody (Novus #NBP1-85886, 1:1000) were used, respectively. β-Actin, probed by a monoclonal anti-β-actin antibody (Sigma #A2228-200UL, 1:1000), served as a loading control for normalization. Absolute quantification of human PCCA and PCCB proteins was performed by LC-MS/MS. Mouse liver was homogenized in 100 mM ammonium bicarbonate and 8 M urea, and spiked with isotopically labeled signature peptides (natural C and N atoms on lysine were replaced by ^13^C and ^15^N isotopes; Thermo Scientific Pierce) for human WT PCCA (AQAVHPGYGFLSENK which does not recognize human PCCA A138T mutant) and PCCB (TVGIVGNQPK or DFFNYLPLSSQDPAPVR) as an internal standard. Protein was reduced, alkylated, and then digested with trypsin (Promega) at 37 °C for 15 h. After desalting, quantification was performed in a Thermo Easy 1200 nano-LC, QE plus Mass Spectrometer, as previously described^1^. The lower limitations of quantification (LLOQs) were 26 and 16 ng/mg tissue for hPCCA and hPCCB proteins, respectively. Protein levels at pmol/mg were calculated from ng/mg based on the molecular weights of hPCCA and hPCCB proteins, which are 80,080 and 59,290 Daltons, respectively.

### PCC enzyme activity assay

PCC enzyme activity was measured using a radiometric activity assay, as previously described^18^. Briefly, cell lysate or the mitochondrial fraction isolated from mouse liver was mixed with PCC substrates, ATP, propionyl CoA, and ^14^C radioisotope-labeled sodium bicarbonate. The reaction mixture was incubated at 37 °C for 15 min, followed by the addition of 5% trichloroacetic acid to stop the reaction, and was then centrifuged at 13,000×*g* for 5 min. The supernatant was collected for quantification of the radioisotope-labeled enzymatic product, methylmalonyl-CoA, using a Microbeta2 scintillation counter (PerkinElmer).

### Quantification of biomarkers in plasma and tissues

To assess the primary disease biomarkers (2MC, C3/C2, 3HP), blood was collected through either submandibular bleeding in study or cardiac puncture upon sacrifice, dispensed into prechilled tubes containing K_2_EDTA and processed for plasma within 15 min of collection. Plasma concentrations of 2MC, C3, C2 for normalization of C3, and 3HP were quantified via LC-MS/MS^40–42^ by Charles River Laboratories (Worcester, MA). Briefly, plasma sample aliquots were mixed at a volume ratio of 1:6 with internal standards containing 3-hydroxypropionic acid-d4, 2-methyl-d3-citric acid, acetyl-d3 L-carnitine, and R-propionyl-d3 carnitine in 95:5 (v:v) acetonitrile:formic acid, evaporated to dryness under nitrogen in a Turbovap set to 45 °C, and reconstituted with hydrochloric acid in 1-butanol followed by incubation at 60 °C for 30 min. The derivatized samples were diluted with 50:50 (v:v) water:acetonitrile (dilution factor = 2). For the analysis of C2 and C3, the diluted samples were further diluted with 80:20 (v:v) water:acetonitrile (total dilution factor = 10). The diluted samples were then run through a Waters HSS T3, 2.5-μm column (30 × 2.1 mm) with 0.1% (v/v) formic acid in water and 0.1% (v/v) formic acid in acetonitrile as mobile phases A and B, respectively, at a flow rate of 0.800 mL/min, and an Applied Biosystems Sciex API-5500 triple-quadrupole mass spectrometer operated in positive ion mode. The LLOQ was 0.5 μM for plasma 2MC, C3, and C2 carnitines, and 25 μM for plasma 3HP assays. Tissue samples (in milligrams) were homogenized in four volumes (in microliters) of water: acetonitrile (80:20, v/v) (dilution factor = 5) and tissue 2MC concentrations were quantified by LC-MS/MS, as described for plasma 2MC. The LLOQ of 2MC in all tissues was 2.5 or 5 nmol/g tissue. All primary disease biomarkers were quantified in a blinded fashion.

Plasma ammonia as the secondary disease biomarker was measured by the clinical analyzer (Randox) following a method reported previously^43^ with an LLOQ of 23.4 μM.

The plasma level of the cardiac biomarker, CTNT, was quantified using an ELISA kit (LSBio Cat# LS-F6996) following the manufacturer’s instructions, with an LLOQ of 7.82 pg/mL.

### Safety evaluations in 3- and 6-month studies in PA mice

Clinical observations were performed and recorded at least five times per week for the 3-month study and at least twice per week for the 6-month study. Detailed clinical observations included, but were not limited to, identification of clinical signs related to general appearance (e.g., skin, fur, changes in eyes, eyeballs, and mucous membranes; presence or absence of discharge), body position and posture (e.g., hunchback posture), autonomic nervous system function (e.g., lacrimation, piloerection, pupil diameter, respiration, excretion), motor coordination, ambulatory abnormalities, reaction to being handled and to environmental stimulation, nervous system (e.g., tremor, convulsion, muscular contractions), changes in exploratory behavior, ordinary behavior (e.g., changes in grooming, headshaking, gyration), abnormal behavior (e.g., autophagia, backward motion, abnormal vocalization), and aggression.

Body weights of all the mice were recorded at least twice per week for the 3-month study and were recorded prior to each dose administration for the 6-month study.

Blood samples were collected into a syringe and then transferred into serum separator tubes and allowed to clot for at least 20 min (not to exceed 1 h), then centrifuged at 5600 × *g* at room temperature for at least 15 min. Alternatively, blood samples were collected into tubes containing K_2_EDTA and were processed to obtain plasma. The resulting serum/plasma samples were frozen on dry ice as soon as possible until stored in a freezer set to maintain −80 °C before shipment to IDEXX (North Grafton, MA) for sample analysis. Clinical chemistry parameters were measured in a blinded manner by a standard chemistry analyzer in IDEXX. The full clinical chemistry panel was assessed in the 3-month study. A select list of liver chemistry parameters was evaluated in the 6-month study.

In the 3-month study, the histopathological evaluation was performed by an experienced veterinary pathologist on a full panel of tissues identified in Supplementary Table 3 from all mice. Tissues were collected, fixed in 10% neutral buffered formalin, and embedded in paraffin, sectioned, mounted on glass slides, and stained with hematoxylin and eosin. A complete gross pathological examination was performed in addition to a detailed microscopic evaluation of all tissues.

In the 3-month study, plasma anti-PEG IgM was quantified using a mouse Anti-PEG IgM ELISA kit (Life Diagnostics #PEGM-1) according to manufacturer’s instruction with modification. Briefly, seven nonzero standards and two sets of three levels of QCs were included. The minimum required dilution of samples was 1:50. The LLOQ was 156.50 units/mL. Absorbance was recorded using a Synergy H1 microplate reader (BioTek).

### Pharmacokinetic and statistical analysis and reproducibility

Mean hPCCA and hPCCB protein concentrations *vs*. nominal time data were used to calculate the pharmacokinetic parameters for hPCCA and hPCCB in the liver by standard noncompartmental methods using Phoenix^®^ WinNonlin^®^ version 8.0 (Certara USA, Inc). *T*_max_ is defined as the time of maximum protein concentration after drug administration, *C*_max_ as maximum protein concentration after drug administration, AUC_(0-inf)_ as the area under the protein concentration/activity versus time curve from time 0 to infinity, and *T*_1/2_ as protein half-life.

All data are expressed as mean ± standard error of the mean (SEM). Values below LLOQ were imputed as half LLOQ. Statistical analyses were performed using GraphPad Prism v.7.01 or SAS version 9.4 software. Means were compared by one-way or two-way ANOVA or the nonparametric equivalent as appropriate. Significant ANOVA findings were followed by Tukey’s or Dunnett’s post hoc test for multiple comparisons. Longitudinal biomarker data from the long-term 3- and 6-month studies were evaluated using a repeated-measures mixed model. Two-tailed *P* values <0.05 were considered statistically significant.

Key findings were successfully replicated in a minimum of two separate experiments.

### Reporting summary

Further information on research design is available in the Nature Research Reporting Summary linked to this article.

## Supplementary information

Supplementary Information

Description of Additional Supplementary Files

Supplementary Data 1

Reporting Summary

## Data Availability

All data generated or analyzed during this study are included in this published article and its supplementary information files. GRCm38 mouse genome was retrieved from GENCODE (https://www.gencodegenes.org/mouse/). Source data are provided with this paper.

## Source data

Source Data
