# Supplementary material for: Dual mRNA therapy restores metabolic function in long-term studies in mice with propionic acidemia
Source: Nat Commun. 2020 Oct 21;11:5339. doi: 10.1038/s41467-020-19156-3 (PMC7578066; doi:10.1038/s41467-020-19156-3)
Supplement: Supplementary file 1 — Supplementary Information [file 41467_2020_19156_MOESM1_ESM.pdf]

Dual mRNA therapy restores metabolic function in long-term studies in mice with propionic acidemia

Jiang et al

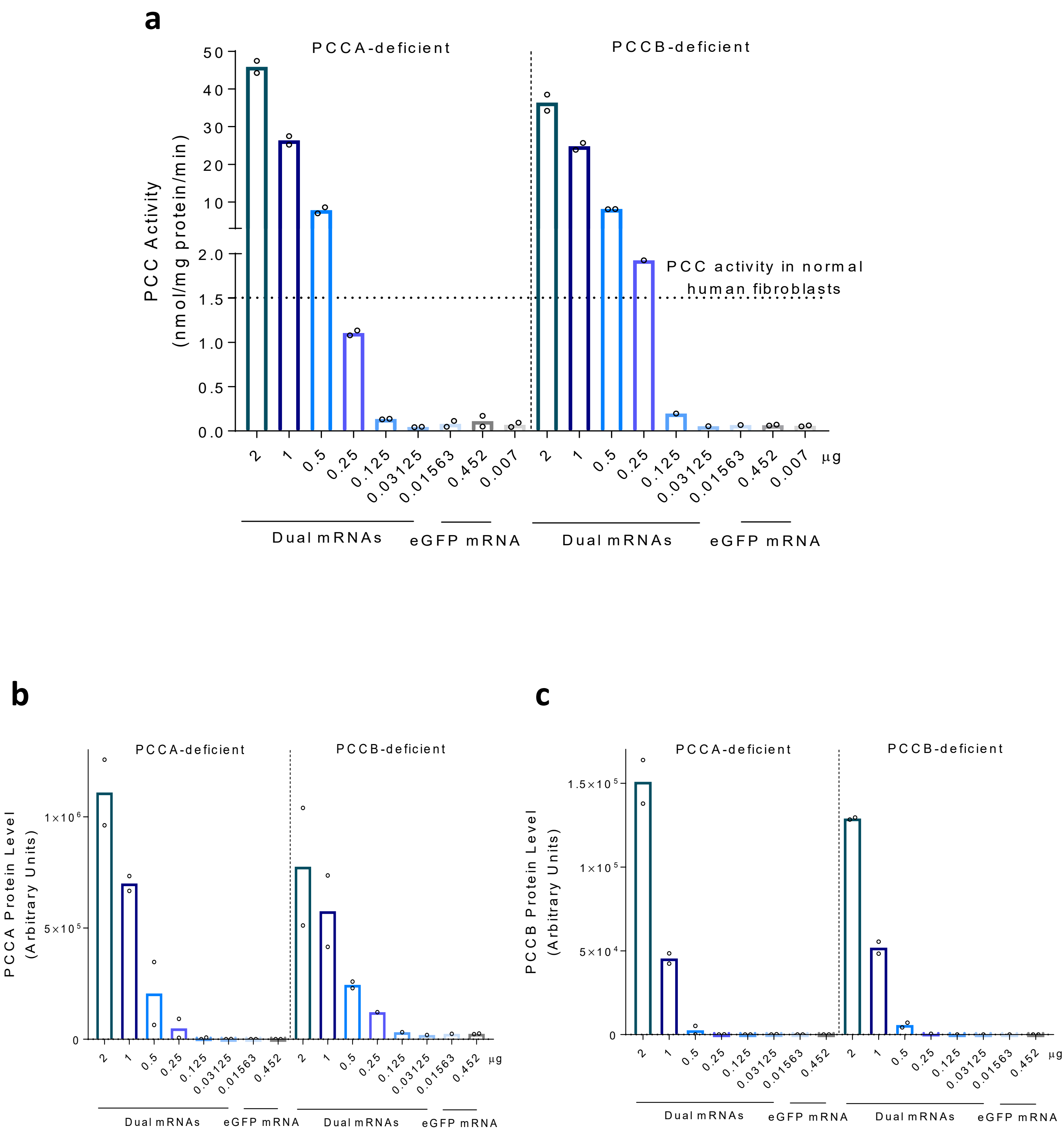

Supplementary Fig. 1

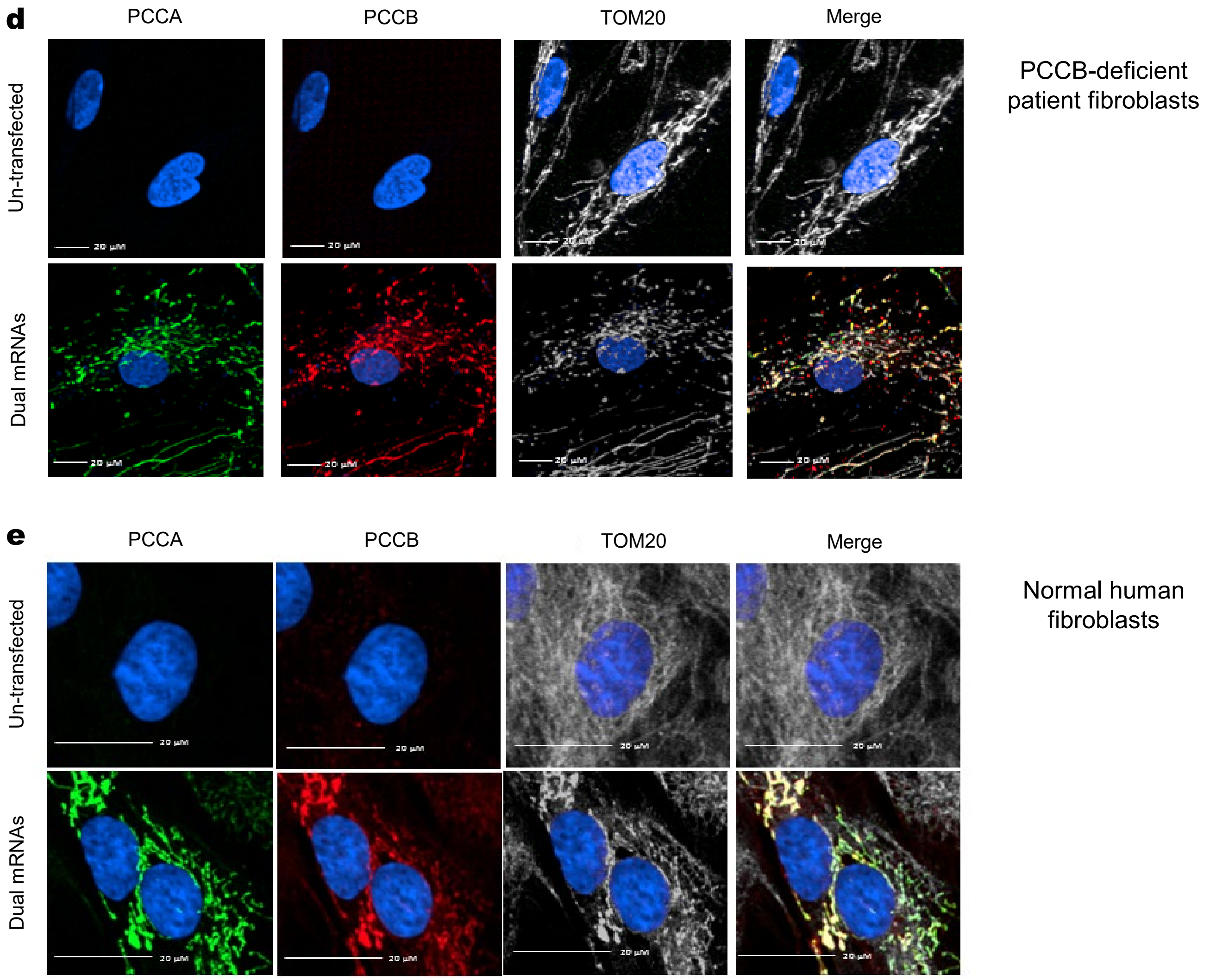

Supplementary Fig. 1. Dose-dependent increases in PCC activity and protein subunits due to dual mRNAs and mitochondrial localization of mRNA-encoded PCC subunits in human fibroblasts. a-c, PA patient-derived fibroblasts were transfected with 0.01563 - 2 µg of total dual mRNAs or 0.007 – 0.452 µg of eGFP mRNA for 24 hours (n=2 per condition). Cells were lysed and mitochondrial matrix fractions were collected to assess PCC enzymatic activity **(a)**, and protein levels of PCCA **(b)** and PCCB **(c)** by capillary electrophoresis. **d-e,** Subcellular localization of dual mRNAs-encoded PCC subunits in PCCB-deficient patient fibroblasts **(d)**, and normal human fibroblasts (ATCC #PCS-201-012) **(e)**. Scale bars are 20 µm. Representative images are shown from n=24 replicates of dual mRNAs or luciferase (Luc) mRNA-transfected cells, and n=12 replicates of un-transfected cells. Endogenous PCCA or PCCB protein was not detected in un-transfected fibroblasts probably due to low protein levels in fibroblasts.

Supplementary Fig. 1 (cont'd)

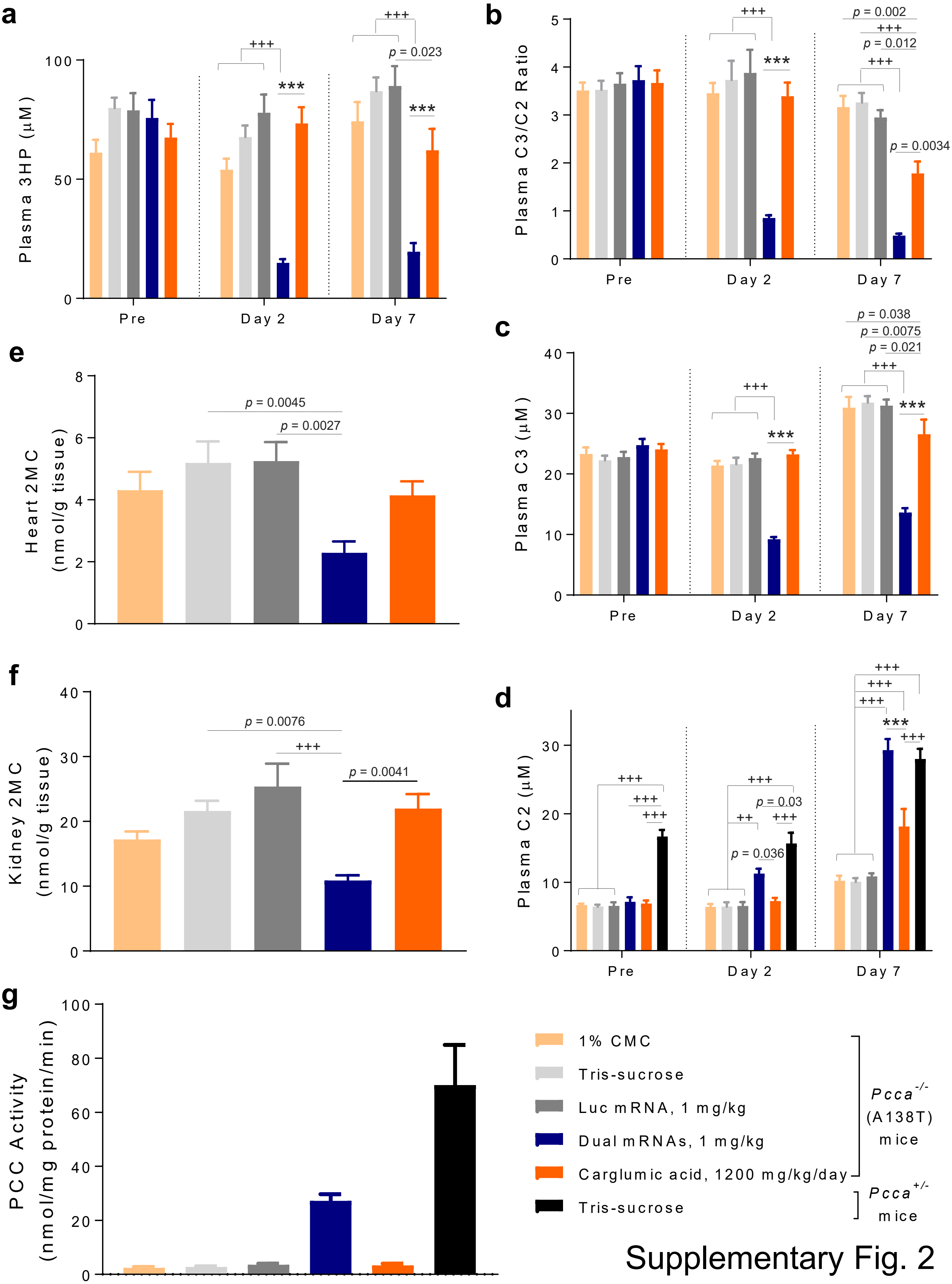

Supplementary Fig. 2

Supplementary Fig. 2. **Substantial reductions in primary disease biomarkers due to restoration of hepatic PCC enzyme by dual mRNAs in contrast to carglumic acid.** PA hypomorphic *Pcca*$^{-/-}$(A138T) female mice were administered a single IV bolus dose of Tris-sucrose buffer (n=11) or 1 mg/kg of dual mRNAs or Luc control mRNA (n=12) encapsulated in LNPs and were sacrificed 7 days post-injection. Additional PA hypomorphic female mice were administered repeat doses of 1% carboxymethyl cellulose (CMC, n=11) or carglumic acid (1200 mg/kg/day, twice daily, n=12) via oral gavage for 7 consecutive days and then sacrificed. Unaffected *Pcca*$^{+/-}$ mice (n=9) were IV injected with Tris-sucrose buffer as control. Mice were bled at study days 0 (pre-treatment), 2 and 7. Plasma 3HP **(a)**, C3/C2 ratio **(b)**, C3 **(c)**, and C2 **(d)** levels, and heart and kidney 2MC concentrations **(e, f)** and PCC activity in liver mitochondria **(g)** at day 7 upon sacrifice were assessed. Biomarker levels in unaffected mice were below lower limitation of quantification (<LLOQ) for plasma 3HP, heart or kidney 2MC, and 0.053 ± 0.005 and 0.881 ± 0.050 for plasma C3/C2 ratio and plasma C3, respectively, at pre-treatment. Following a single dose of dual mRNAs, plasma C3 was decreased **(c)** whereas plasma C2 was increased trending towards the level of unaffected control mice **(d)** possibly due to the restored free carnitine for C2. Increased concentrations of plasma C3 and C2 observed at sacrifice on Day 7 compared to those on Days 0 and 2 could be due to terminal cardiac puncture *vs.* in-life submandibular bleeding. Data are shown as mean ± SEM. *p*-values were obtained from Tukey's post-hoc pairwise multiple comparison test following a one-way or two-way ANOVA, and are provided in the source data. *** $p<0.001$ comparing dual mRNA therapy with carglumic acid. ++ $p<0.01$, +++ $p<0.001$ comparing dual mRNAs or carglumic acid group with control groups (1% CMC, Tris-sucrose and Luc mRNA). Pre, pre-treatment on Day 0.

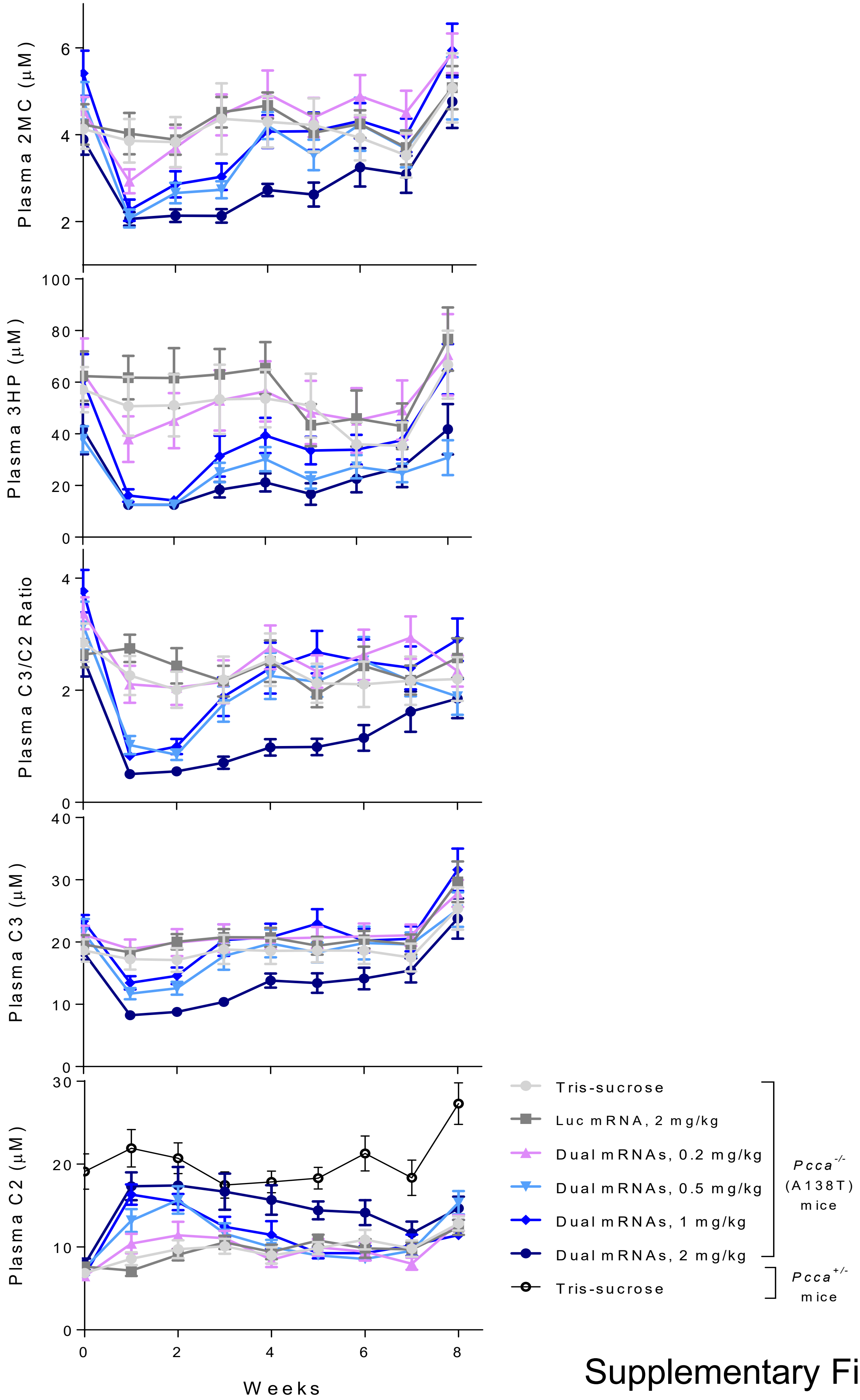

Supplementary Fig. 3

Supplementary Fig. 3. Single administration dose-range-finding study. PA hypomorphic mice of mixed gender were administered a single IV bolus injection of dual mRNAs at 0.2, 0.5, 1 or 2 mg/kg, Luc mRNA at 2 mg/kg, or Tris-sucrose buffer (n=11 mice/group). Blood was collected weekly throughout the 8-week study. Plasma primary disease biomarkers, 2MC, 3HP, C3/C2 ratio and C3 were quantified, together with plasma C2. Biomarker levels in age-matched unaffected mice included in the study (n=12) were <LLOQ for plasma 2MC or 3HP, and <0.1 and <2.3 mM for plasma C3/C2 ratio and C3, respectively, across all time points. Data shown as mean ± SEM. The dose levels and regimen for the 3-month study in PA hypomorphic mice were determined based on biomarker responses observed in this dose-range-finding study.

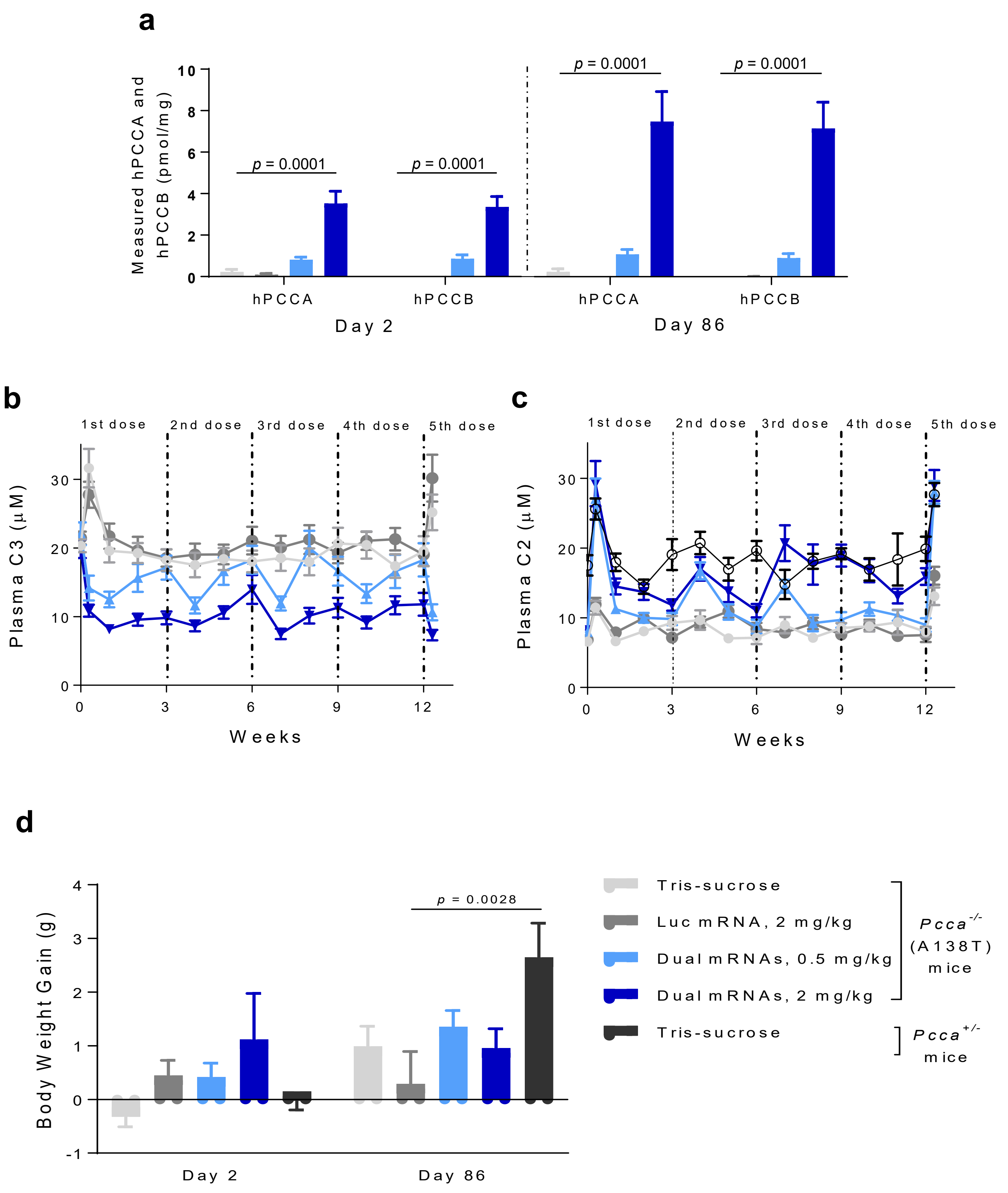

Supplementary Fig. 4

e

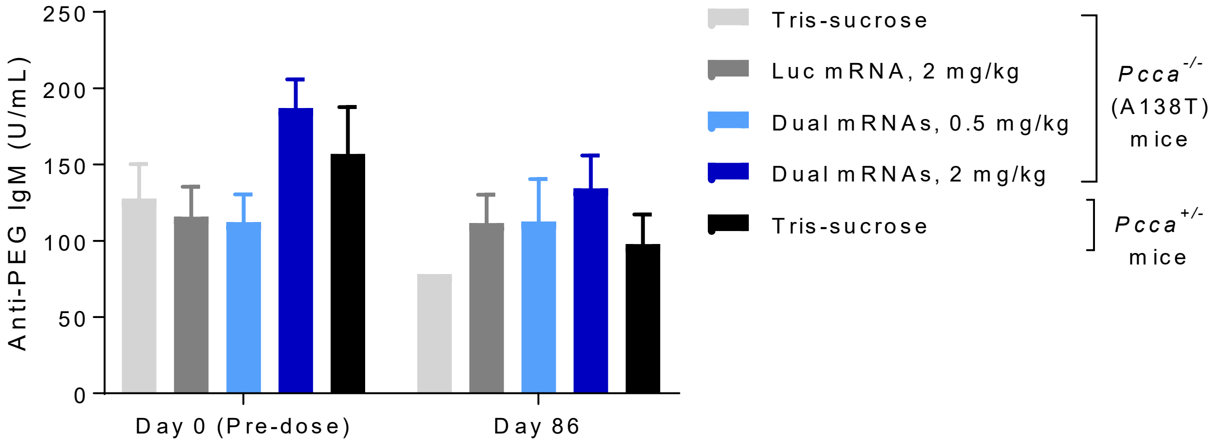

f

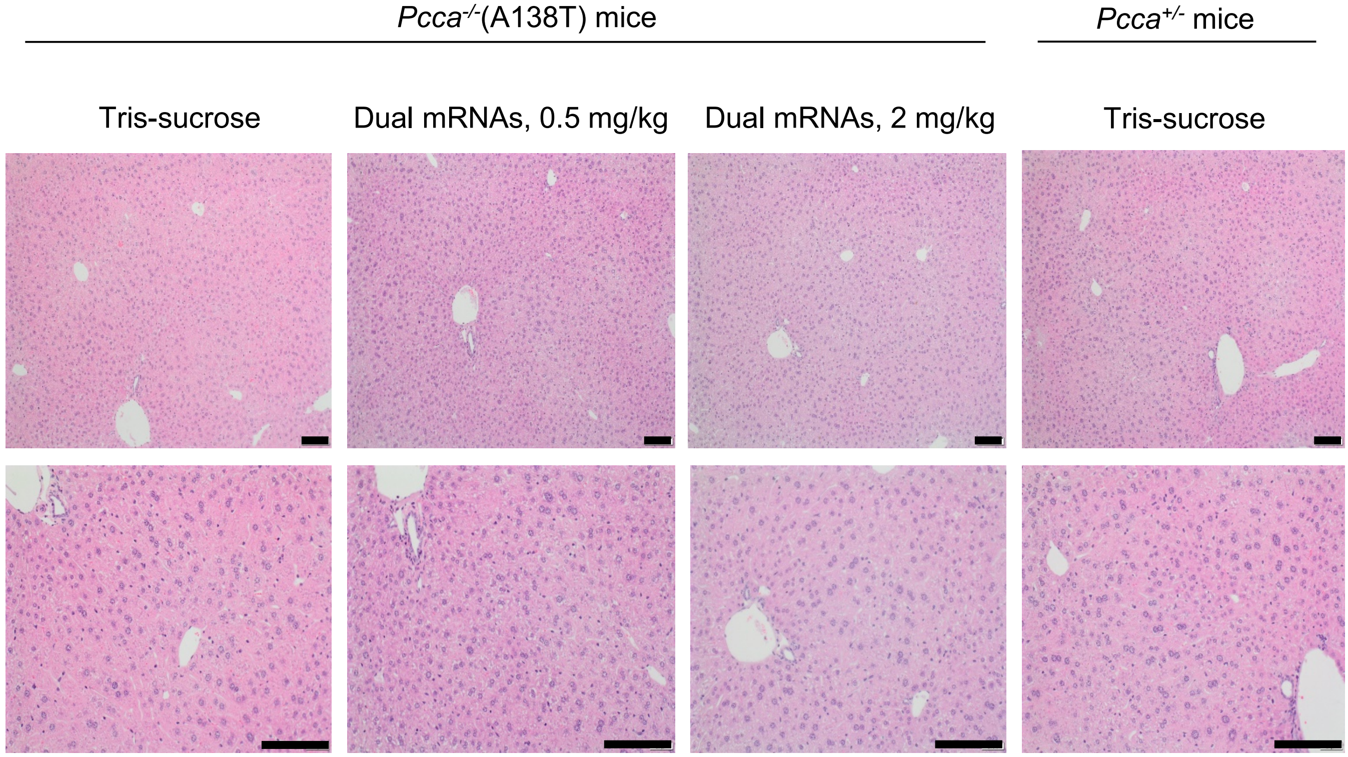

Supplementary Fig. 4. 3-month repeat-dose study in PA hypomorphic mice. PA hypomorphic mice of mixed gender were administered 0.5 or 2 mg/kg dual mRNAs or 2 mg/kg Luc control mRNA or Tris-sucrose buffer (n=12/group/sacrifice time point) every 3 weeks for 12 weeks. Mice were sacrificed 2 days after the first and last doses (Days 2 and 86, respectively). mRNA-encoded hPCCA and hPCCB protein levels were quantified by LC-MS/MS at each sacrifice **(a)**. Plasma C3 **(b)** and C2 **(c)** were assessed weekly throughout the 3-month study. Plasma C3 levels in unaffected mice were <2.0 mM for all time points. **d,** Body weights were recorded at each sacrifice and compared with baseline before treatment. **e,** Plasma anti-PEG IgM levels were assessed on Days 0 (pre-dose) and 86. **f,** Histopathology of liver was examined and representative Day 86 hematoxylin and eosin images of unaffected and PA hypomorphic mice are presented. Scale bars are 50 µm. All data shown as mean ± SEM. *p*-values were obtained from Tukey's or Dunnett's post-hoc pairwise multiple comparison test following a two-way ANOVA.

Supplementary Fig. 4 (cont'd)

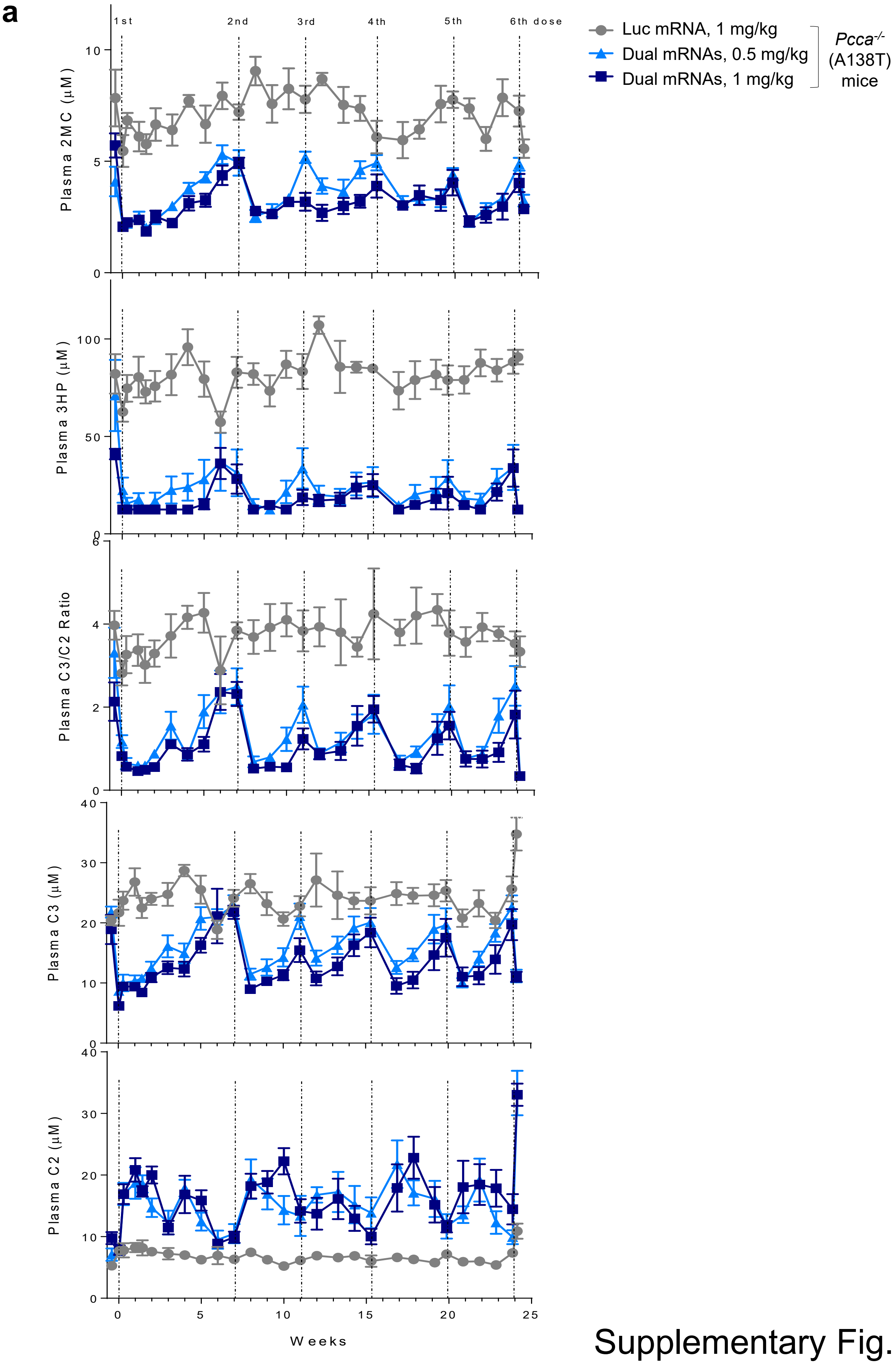

Supplementary Fig. 5

b

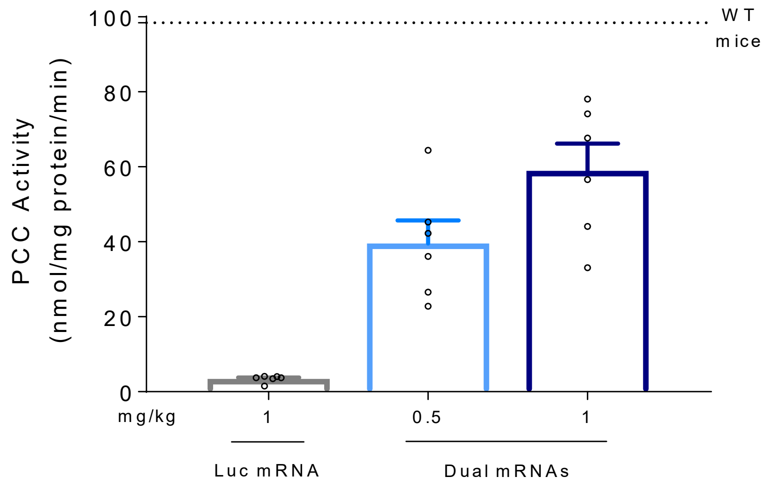

c

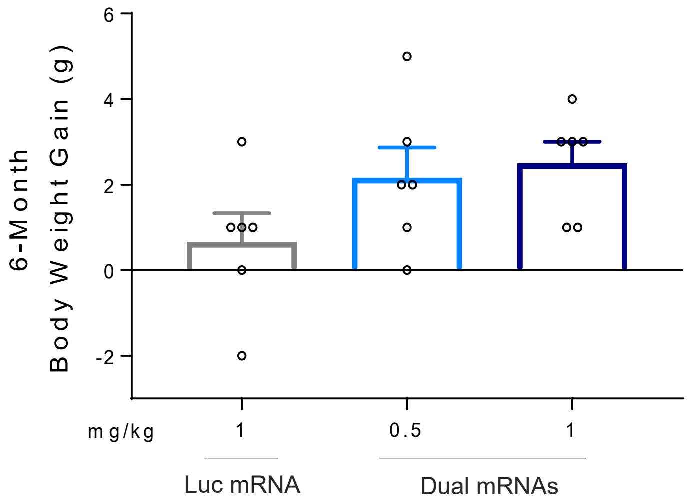

Supplementary Fig. 5. Long-term pharmacology in 6-month repeat-dose study. PA hypomorphic female mice received a total of 6 IV doses of 0.5 or 1 mg/kg dual mRNAs or 1 mg/kg Luc control mRNA (n=6/group) at weeks 0, 7, 11, 15, 20 and 24. Mice were sacrificed 2 days after the 6th dose and serially bled throughout the study. Age-matched untreated female WT mice (n=6) were added to the end of study at sacrifice. **a,** Plasma primary disease biomarkers, 2MC, 3HP, C3/C2 ratio and C3 were evaluated, together with plasma C2. Biomarker levels in WT mice were <LLOQ for plasma 2MC and 3HP, and 0.06 ± 0.01, 1.54 ± 0.49 mM and 23.15 ± 3.74 mM for plasma C3/C2 ratio, C3 and C2, respectively. All *p*-values were <0.001 from a repeated-measures mixed model of change from baseline across all time points and for time points after each dose administration. Increased concentrations of plasma C3 and C2 observed at the end of the study compared to other collection time points could be due to terminal cardiac puncture *vs.* in-life submandibular bleeding. **b,** PCC activity in liver mitochondria was measured upon sacrifice. The PCC activity for n=6 untreated age-matched WT mice was 98.43 ± 5.36 nmol/mg protein/min. **c,** Body weights were recorded at sacrifice and compared with baseline before treatment. Data are presented as mean ± SEM.

Supplementary Fig. 5 (cont'd)

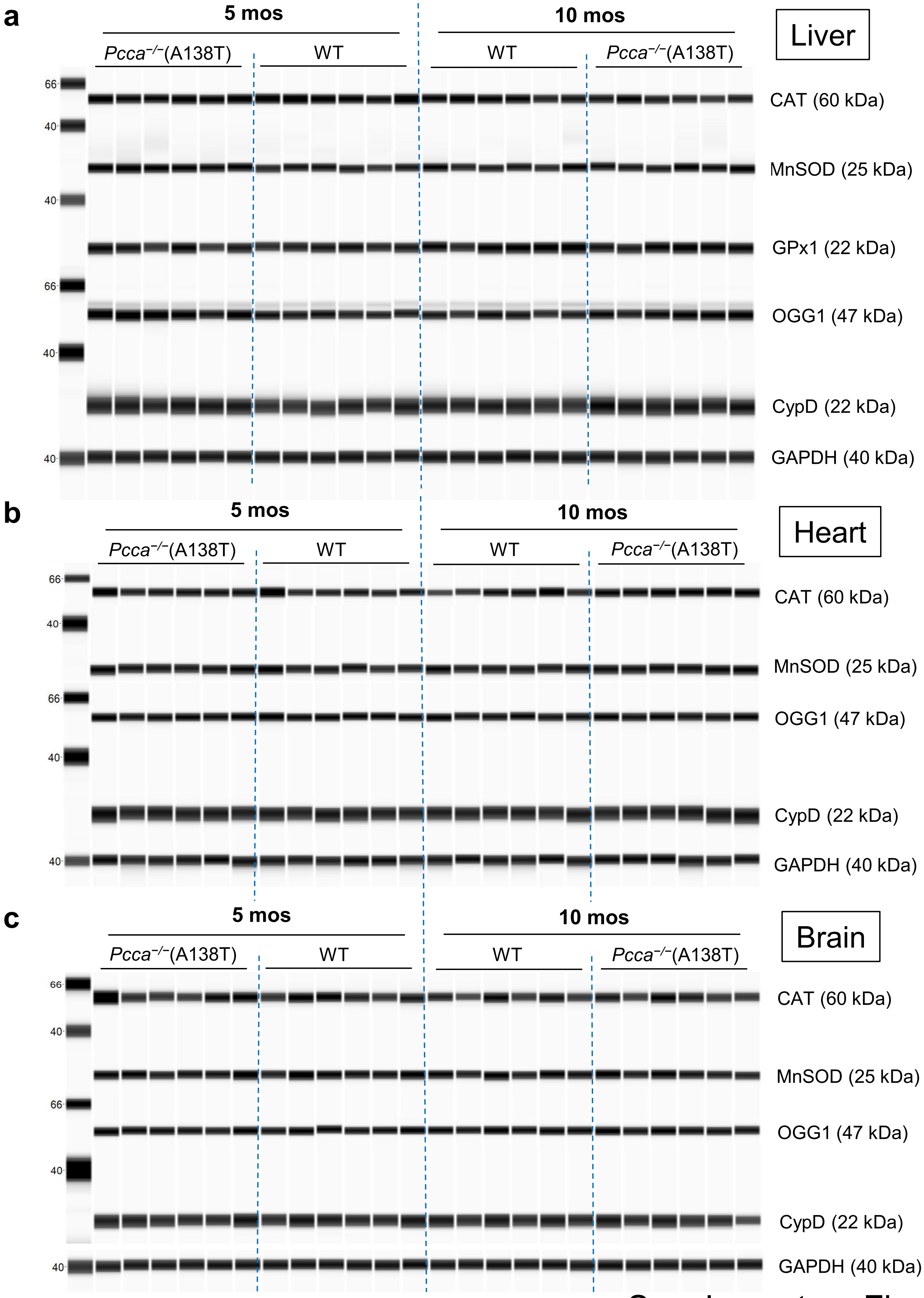

Supplementary Fig. 6

d

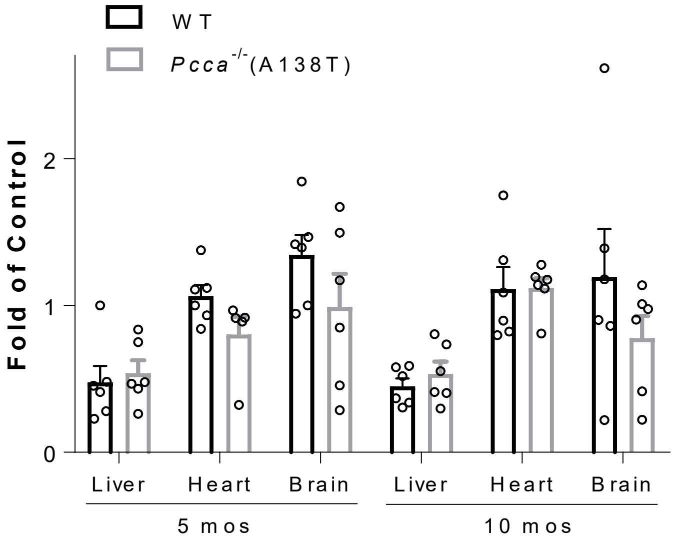

e

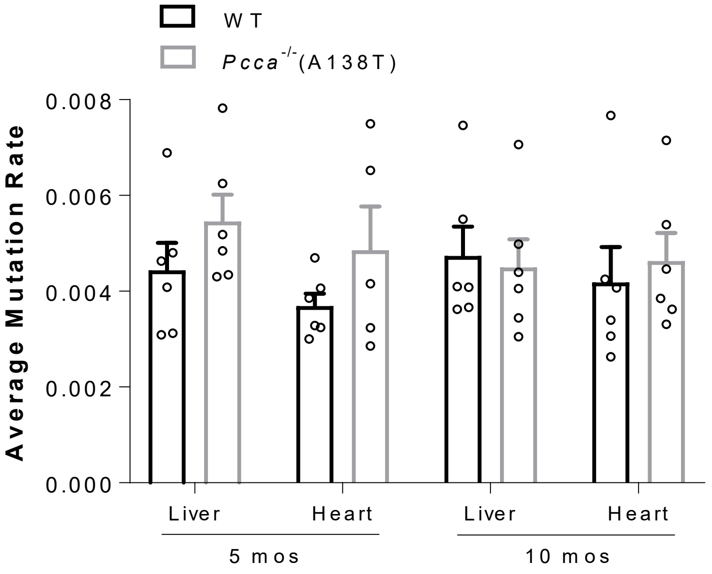

Supplementary Fig. 6. Mild changes in oxidative protein markers and no alteration in mitochondrial genome in PA hypomorphic mice. Oxidative protein markers and mitochondrial genome were assessed in 5- and 10-month-old WT and PA hypomorphic mice of mixed gender (n=6/genotype/age/organ). **a-c,** Levels of catalase (CAT), manganese superoxide dismutase (MnSOD), glutathione peroxidase 1 (GPx1), 8-oxoguanine DNA glycosylase 1 (OGG1) and cyclophilin D (CypD) in liver **(a)**, heart **(b)** and brain **(c)** were semi-quantified via capillary electrophoresis. GAPDH served as a loading control. Representative bandviews of each protein are shown. **d-e,** mitochondrial DNA (mtDNA) assessment. **d,** mtDNA copy numbers in liver, heart and brain. Data from one out of two primer sets are presented as representative. **e**, Aggregate mutation rate of mtDNA in liver and heart. All data shown as mean ± SEM.

Supplementary Fig. 6 (cont'd)

Supplementary Table 1. Full codon-optimized hPCCA and hPCCB open reading frame sequences.

hPCCA mRNA sequence:

AUGGCCGGCUUCUGGGUCGGCACAGCCCCUCUGGUGGCAGCCGGCAGAAGAGGACGGUGG
CCUCCCCAGCAACUGAUGCUGAGCGCCGCCCUGAGAACCCUGAAGCACGUGCUGUACUAC
AGCAGACAGUGCCUGAUGGUGAGCAGAAAUCUGGGCAGCGUGGGGUACGAUCCCAACGA
GAAGACCUUCGAUAAGAUUCUGGUCGCGAAUAGAGGCGAGAUCGCCUGCAGGGUGAUCA
GAACCUGCAAGAAGAUGGGCAUCAAGACCGUGGCCAUCCAUUCGGACGUCGACGCGAGCA
GCGUUCACGUGAAGAUGGCAGACGAGGCCGUGUGCGUGGGACCCGCCCCGACCAGCAAGA
GCUACCUGAACAUGGACGCCAUCAUGGAGGCCAUCAAGAAGACCCGCGCUCAAGCCGUGC
ACCCGGGCUACGGCUUUCUGAGCGAGAACAAGGAAUUCGCCAGGUGUCUCGCCGCCGAGG
ACGUAGUCUUCAUCGGCCCUGAUACGCACGCGAUCCAGGCCAUGGGCGACAAGAUCGAGA
GCAAACUGCUGGCCAAGAAAGCAGAAGUCAACACCAUCCCCGGCUUCGACGGCGUGGUGA
AGGACGCCGAAGAGGCUGUCCGCAUCGCCAGAGAGAUCGGCUACCCUGUGAUGAUAAAGG
CUAGCGCUGGAGGUGGCGGAAAGGGCAUGAGAAUCGCCUGGGACGACGAGGAGACUAGA
GACGGCUUCAGACUGUCCUCCCAGGAGGCCGCCAGCUCCUUCGGAGACGACAGACUGCUG
AUCGAGAAGUUCAUCGACAACCCCAGACACAUCGAAAUCCAGGUGCUCGGUGACAAGCAC
GGGAACGCCCUGUGGCUGAACGAGAGAGAGUGCAGCAUCCAGAGAAGAAACCAGAAGGU
GGUGGAGGAGGCGCCGAGCAUCUUUCUGGACGCGGAGACAAGGAGAGCGAUGGGCGAAC
AGGCCGUCGCCCUAGCAAGAGCCGUGAAGUACUCCAGUGCCGGAACCGUCGAGUUUCUUG
UCGACAGCAAGAAGAAUUUCUACUUCCUGGAGAUGAACACCAGGCUGCAGGUGGAGCAU
CCCGUGACAGAGUGCAUCACUGGACUGGAUCUGGUGCAGGAGAUGAUCAGGGUGGCCAA
GGGCUAUCCCCUGAGACACAAGCAGGCCGACAUCAGAAUCAACGGCUGGGCCGUGGAGUG
CAGAGUGUACGCCGAGGACCCCUACAAGAGCUUCGGCCUGCCCAGCAUCGGCAGACUGAG
CCAGUACCAGGAGCCCCUGCACCUGCCCGGCGUGAGAGUGGACAGCGGCAUCCAACCGGG
GAGCGAUAUCAGCAUCUACUACGACCCCAUGAUCAGCAAGCUGAUAACCUACGGCAGCGA
CAGAACCGAGGCCCUGAAGAGAAUGGCCGACGCCCUGGACAACUACGUGAUCAGAGGCGU
GACCCACAACAUCGCCCUGCUGAGAGAGGUGAUCAUCAACUCGAGGUUCGUGAAAGGCGA
CAUCAGCACCAAGUUCCUGAGCGACGUGUAUCCCGACGGAUUCAAAGGUCACAUGCUGAC
CAAGAGCGAGAAGAACCAGCUGCUGGCCAUCGCCUCAUCCCUGUUCGUGGCCUUCCAGCU
GAGAGCCCAGCACUUCCAGGAGAACAGCAGAAUGCCCGUGAUCAAGCCCGACAUCGCCAA
CUGGGAGCUGAGCGUGAAGCUGCACGACAAGGUGCACACUGUCGUUGCCAGCAACAACGG
CUCCGUGUUCAGCGUAGAGGUGGACGGAUCUAAGCUGAACGUGACCUCCACCUGGAACCU
GGCAAGCCCUCUCCUGUCAGUGAGCGUGGACGGCACCCAGAGAACCGUGCAGUGUCUGUC
CCGCGAGGCCGGCGGAAACAUGAGCAUCCAGUUCCUGGGCACCGUGUACAAGGUGAACAU
CCUGACCAGACUGGCCGCCGAGCUGAACAAGUUCAUGCUGGAGAAAGUGACGGAGGAUAC
CAGCUCCGUGCUGAGAAGCCCCAUGCCCGGAGUGGUGGUGGCCGUUUCCGUGAAACCUGG
UGACGCCGUGGCCGAGGGGCAAGAGAUCUGCGUGAUCGAGGCCAUGAAGAUGCAGAAUU
CCAUGACCGCCGGAAAGACCGGCACCGUCAAAUCAGUGCACUGCCAGGCGGGCGACACAG
UGGGUGAGGGCGACCUGCUGGUGGAGCUGGAGUGAUAAUAG

hPCCB mRNA sequence:

AUGGCGGCGGCAUUACGGGUGGCGGCGGUCGGGGCAAGGCUCAGCGUGCUGGCCAGCGGC
CUGAGAGCCGCCGUGAGAAGCCUGUGCAGCCAGGCCACCAGCGUGAACGAGAGAAUCGAG
AACAAGAGAAGAACCGCCCUGCUGGGCGGCGGCCAGAGAAGAAUCGACGCCCAGCACAAG
AGAGGCAAGCUGACCGCCAGAGAGAGAAUCAGCCUGCUGCUGGACCCCGGCAGCUUCGUG
GAGAGCGACAUGUUCGUGGAGCACAGGUGCGCCGACUUCGGCAUGGCCGCCGACAAGAAC
AAGUUCCCCGGCGACAGCGUGGUGACCGGCAGAGGCAGAAUCAACGGCAGACUGGUGUAC
GUGUUCAGCCAGGACUUCACCGUGUUCGGCGGCAGCCUGAGCGGCGCCCACGCCCAGAAG
AUCUGCAAGAUCAUGGACCAGGCCAUCACCGUGGGCGCGCCCGUGAUCGGCCUGAACGAC
AGCGGCGGCGCCAGAAUCCAGGAGGGCGUGGAGAGCCUGGCCGGCUACGCCGACAUCUUC
CUGAGAAACGUGACCGCCAGCGGCGUGAUCCCACAGAUCAGCCUGAUCAUGGGCCCCUGC
GCCGGCGGCGCCGUGUACAGCCCCGCCCUGACCGACUUCACCUUCAUGGUGAAGGACACC
AGCUACCUGUUCAUCACCGGCCCCGACGUGGUGAAGAGCGUGACCAACGAGGACGUGACC
CAGGAGGAGCUGGGCGGCGCCAAGACCCACACCACCAUGAGCGGCGUGGCCCACAGAGCC
UUCGAGAACGACGUGGACGCCCUGUGCAACCUGAGAGACUUCUUCAACUACCUGCCCCUG
AGCAGCCAGGACCCCGCGCCCGUGAGAGAGUGCCACGACCCCAGCGACAGACUGGUGCCC
GAGCUGGACACCAUCGUGCCCCUGGAGAGCACCAAGGCCUACAACAUGGUGGACAUCAUC
CACAGCGUGGUGGACGAGAGAGAGUUCUUCGAGAUCAUGCCCAACUACGCCAAGAACAUC
AUCGUGGGCUUCGCCAGAAUGAACGGCAGAACCGUGGGCAUCGUGGGCAACCAGCCCAAG
GUGGCCAGCGGCUGCCUGGACAUCAACAGCAGCGUGAAGGGCGCCAGAUUCGUGAGAUUC
UGCGACGCCUUCAACAUCCCUCUGAUCACCUUCGUGGACGUGCCCGGCUUCCUGCCCGGC
ACCGCCCAGGAGUACGGCGGCAUCAUCAGACACGGCGCCAAGCUGCUGUACGCCUUCGCC
GAGGCCACCGUGCCCAAGGUGACCGUGAUCACCAGAAAGGCCUACGGCGGCGCCUACGAC
GUGAUGAGCAGCAAGCACCUGUGCGGCGACACCAACUACGCCUGGCCCACCGCCGAGAUC
GCCGUGAUGGGCGCCAAGGGCGCCGUGGAGAUCAUCUUCAAGGGCCACGAGAACGUGGAG
GCCGCCCAGGCCGAGUACAUCGAGAAGUUCGCCAACCCCUUCCCCGCCGCCGUGAGAGGC
UUCGUGGACGACAUCAUCCAGCCCAGCAGCACCAGAGCCAGAAUCUGCUGCGACCUGGAC
GUGCUGGCCAGCAAGAAGGUGCAGAGACCCUGGAGAAAGCACGCCAACAUCCCUCUGUGA
UAAUAG

Note: An alternative PCCB sequence with high identity was used in some preliminary studies.

Supplementary Table 2. Selected clinical chemistry parameters in the 6-month study.

Genotype	*Pcca*$^{-/-}$(A138T)			WT
Test Article	Luc mRNA	Dual mRNAs		N/A
Dose (mg/kg)	1	0.5	1	N/A
n	6	6	5-6	4-5
ALT (U/L)	133.83 ± 17.24	51.00 ± 7.66	43.50 ± 6.53	65.60 ± 9.49[a]
AST (U/L)	115.17 ± 10.00	76.83 ± 7.83	79.33 ± 9.89	103.60 ± 14.15[a]
GGT (U/L)	0 ± 0	0 ± 0	0 ± 0[b]	0 ± 0[c]
Triglycerides (mg/dL)	181.33 ± 53.08	145.00 ± 11.86	172.80 ± 11.98[b]	347.25 ± 27.22[c]
Cholesterol (mg/dL)	141.17 ± 6.22	125.67 ± 6.00	135.00 ± 8.75[b]	182.75 ± 40.20[c]
Bile Acids (µmol/L)	5.18 ± 0.28	5.97 ± 1.43	5.26 ± 1.94[b]	6.33 ± 1.27[c]
Albumin (g/dL)	2.83 ± 0.088	3.03 ± 0.092	3.12 ± 0.037[b]	2.90 ± 0.11[c]

Data are presented as mean ± SEM. a, one outlier was excluded from this table. b, n = 5 due to volume shortage. c, n= 4 due to volume shortage.

Supplementary Table 3. Tissues collected for histopathology analysis in the 3-month study.

Artery, aorta	Gland, pituitary	Lymph node, mandibular	Testis
Body cavity, nasal	Gland, prostate	Lymph node, mesenteric	Thymus
Bone marrow smear	Gland, salivary	Muscle, skeletal	Tongue
Bone marrow	Gland, seminal vesicle	Nerve, optic	Trachea
Bone, femur	Gland, thyroid	Nerve, sciatic	Urinary bladder
Bone, sternum	Gross lesions/masses	Ovary	Uterus
Brain	Gut-associated lymphoid tissue	Pancreas	Vagina
Cervix	Heart	Site, Administration	
Epididymis	Kidney	Skin	
Esophagus	Large intestine, cecum	Small intestine, duodenum	
Eye	Large intestine, colon	Small intestine, ileum	
Gland, adrenal	Large intestine, rectum	Small intestine, jejunum	
Gland, harderian	Larynx	Spinal cord	
Gland, mammary	Liver	Spleen	
Gland, parathyroid	Lung	Stomach	

Supplementary Table 4. Summary of dual mRNAs-related microscopic findings in liver.

Day 2					
Genotype	*Pcca*$^{-/-}$(A138T)				*Pcca*$^{+/-}$
Test Article	Tris-sucrose	Luc mRNA	Dual mRNAs		Tris-sucrose
Dose (mg/kg)	0	2	0.5	2	0
n	10	10	10	10	8
Increased Mitosis	2 (20.0%)	0 (0%)	1 (10.0%)	3 (30.0%)	0 (0%)
Minimal	2 (20.0%)	0 (0%)	1 (10.0%)	2 (20.0%)	0 (0%)
Mild	0 (0%)	0 (0%)	0 (0%)	1 (10.0%)	0 (0%)
**Day 86**					
Genotype	*Pcca*$^{-/-}$(A138T)				*Pcca*$^{+/-}$
Test Article	Tris-sucrose	Luc mRNA	Dual mRNAs		Tris-sucrose
Dose (mg/kg)	0	2	0.5	2	0
n	11	12	11	10	10
Infiltration mixed cells perivascular	1 (9.1%)	8 (66.7%)	4 (36.4%)	4 (40.0%)	0 (0%)
Minimal	0 (0%)	3 (25.0%)	2 (18.2%)	3 (30.0%)	0 (0%)
Mild	1 (9.1%)	5 (41.7%)	2 (18.2%)	1 (10.0%)	0 (0%)

Data presented as n (%).

Supplementary Table 5. Summary of changes in oxidative protein markers in liver, heart and brain of *Pcca*$^{-/-}$(A138T) mice as compared to WT mice.

	5 mos (n = 6 / genotype / organ)			10 mos (n = 6 / genotype / organ)		
	Liver	Heart	Brain	Liver	Heart	Brain
Catalase	- [a]	- [b]	-	0.88 (p = 0.0089)	1.26 (p = 0.049)	-
MnSOD	1.33 (p = 0.0007)	-	-	1.21 (p = 0.038)	-	-
GPx1	-	ND	ND	-	ND	ND
OGG1	1.46 (p = 0.0003)	-	-	1.38 (p = 0.0098)	-	-
CypD	1.19 (p = 0.014)	-	-	1.22 (p = 0.0002)	-	-

Levels of oxidative protein markers in liver, heart and brain of *Pcca*$^{-/-}$(A138T) mice are presented as ratios relative to those in WT mice. a: no significant difference between genotypes. b: Ratio of cardiac Catalase levels in 5-month-old *Pcca*$^{-/-}$(A138T) to WT mice was 1.23 (p = 0.0121) when 1 outlier was removed from the WT group. ND: not detectable. *P* values were obtained from the paired two-tailed t-test.

Supplementary Methods:

Assessment of oxidative markers by capillary electrophoresis

At sacrifice, tissues were harvested and homogenized in a homogenization buffer containing 20 mM Tris (pH 7.5), 150 mM NaCl, 1 mM EDTA, 1 mM EGTA and 1% Triton X-100 supplemented with protease inhibitors. Homogenates were centrifuged at 18,000 *g* for 30 minutes to obtain the supernatants. Semi-quantitative analysis of oxidative protein markers, catalase (CAT), manganese superoxide dismutase (MnSOD), glutathione peroxidase 1 (GPx1), 8-oxoguanine DNA glycosylase 1 (OGG1) and cyclophilin D (CypD)[1,2], was achieved by capillary electrophoresis (Wes, ProteinSimple) based on manufacturer's recommendations. For detection in liver, heart and brain, a protein concentration of 0.1 – 1 mg/ml was loaded, and a polyclonal anti-CAT antibody (Abcam #ab52477, 1:200 – 1:1500), a polyclonal anti-MnSOD antibody (Enzo #ADI-SOD-110, 1:500 – 1:1000), a polyclonal anti-GPx1 antibody (Abcam #ab22604, 1:200 – 1:500), a polyclonal anti-OGG1 antibody (Proteintech #15125-1-AP, 1:500 – 1:1000) and a monoclonal anti-CypD antibody (Abcam #ab110324, 1:250 – 1:1000) were used. GAPDH, probed by a monoclonal anti-GAPDH antibody (Abcam #ab8245, 1:250), served as a loading control for normalization.

Quantification of mitochondrial DNA (mtDNA) copy numbers

mtDNA copy numbers were quantified as previously described[1]. Briefly, DNA was extracted using Promega Maxwell RSC Tissue DNA Kit (#AS1610). Primers for the mitochondrial 12S gene are: 5′-CCTCTTAGGGTTGGTAAATTTCG-3′ or 5′-CAGCCTATATACCGCCATCTTC-3′ (forward), and 5′-CGAAGATAATTAGTTTGGGTTAATCG-3′ or 5′-TTGGCTACACCTTGACCTAAC-3′ (reverse). Primers for the nuclear Atp5b gene are: 5′-

AAGAGCACGGGTCGTGAG-3′ or 5′- CAGAACAGTCAGTGGAGGTTAG-3′ (forward), and 5′-TGAGCTCTCGCTTGATATGG-3′ or 5′-GGGCTTGAGTGGTGGTTTA-3′ (reverse). qPCR analysis was performed using Kapa SYBR Fast Master Mix (#KK4601) in ABI QuantStudio 7 flex instrument with cycling times as per Kapa's recommendations. The relative mtDNA copy number of each mouse was calculated using the $2^{-\Delta\Delta Ct}$ method, and was compared to that of one 5-month-old WT mouse selected as control. The resultant folds of control were further compared between genotypes across ages and organs using the two-way ANOVA Sidak's multiple comparison test.

mtDNA sequencing for mutation detection

mtDNA was enriched from frozen tissue samples through the use of QIAprep Spin Miniprep Kits (Qiagen #27106) as previously detailed[3]. Purified materials then served as input in library construction using Illumina's DNA prep (Illumina #20018704) as per the manufacturer's recommendations. Libraries were barcoded in the process of library construction (Illumina #20018708). Sequencing libraries were assessed for quantity and quality. Libraries were subsequently pooled and sequenced on an Illumina MiSeq instrument as a paired-end 2X150bp run. Quality of the resulting data was verified using a combination of FastQC[4] and multiQC[5]. Reads were aligned to the GRCm38 mouse genome (https://www.gencodegenes.org/mouse/) using the STAR aligner[6] with parameters appropriate for genomic DNA alignment. Mutations on the small mitochondrial genome were counted using a tool for evaluation of mutation rates, originally developed for the study of nucleic acid adducts (ShapeMapper 2[7]) and adapted for its sensitivity to high-quality mutations. Further analysis was conducted using a custom python script.

Averaged mutation rates between genotypes were compared using the two-way ANOVA Sidak's multiple comparison test.

Supplementary References:

1. Gallego-Villar, L. *et al.* In Vivo Evidence of Mitochondrial Dysfunction and Altered Redox Homeostasis in a Genetic Mouse Model of Propionic Acidemia: Implications for the Pathophysiology of This Disorder. *Free Radic. Biol. Med.* **96**, 1-12 (2016).
2. Rivera-Barahona, A. *et al.* Treatment With Antioxidants Ameliorates Oxidative Damage in a Mouse Model of Propionic Acidemia. *Mol. Genet. Metab.* **122**, 43-50 (2017).
3. Quispe-Tintaya, W. *et al.* Fast Mitochondrial DNA Isolation From Mammalian Cells for Next-Generation Sequencing. *Biotechniques* **55**, 133-6 (2013).
4. Andrews, S. FastQC: A Quality Control Tool for High Throughput Sequence Data. Available online at: http://www.bioinformatics.babraham.ac.uk/projects/fastqc (2010).
5. Ewels, P., Magnusson, M., Lundin, S., & Käller, M. MultiQC: Summarize Analysis Results for Multiple Tools and Samples in a Single Report. *Bioinformatics* **32**, 3047–3048 (2016).
6. Dobin, A. *et al*. STAR: Ultrafast Universal RNA-Seq Aligner. *Bioinformatics* **29**, 15–21 (2013).
7. Busan, S., & Weeks, K. M. Accurate Detection of Chemical Modifications in RNA by Mutational Profiling (MaP) with ShapeMapper 2. *RNA* **24**, 143–148 (2018).
