# Supplementary material for: Dual mRNA therapy restores metabolic function in long-term studies in mice with propionic acidemia
Source: Nat Commun. 2020 Oct 21;11:5339. doi: 10.1038/s41467-020-19156-3 (PMC7578066; doi:10.1038/s41467-020-19156-3)
Supplement: Supplementary file 2 — Description of Additional Supplementary Files [file 41467_2020_19156_MOESM2_ESM.pdf]

Description of Additional Supplementary Files

Title: Supplementary Data 1.

Description: Selected serum clinical chemistry parameters in the 3-month study in PA hypomorphic mice. Data are presented as mean ± SEM. N/A, not applicable.
