# Supplementary material for: Dual mRNA therapy restores metabolic function in long-term studies in mice with propionic acidemia
Source: Nat Commun. 2020 Oct 21;11:5339. doi: 10.1038/s41467-020-19156-3 (PMC7578066; doi:10.1038/s41467-020-19156-3)
Supplement: Supplementary file 4 — Reporting Summary [file 41467_2020_19156_MOESM4_ESM.pdf]

nature research

Corresponding author(s): Lin T. Guey, lin.guey@modernatx.com; Paolo G.V. Martini, paolo.martini@modernatx.com

Last updated by author(s): Sep 8, 2020

Reporting Summary

Nature Research wishes to improve the reproducibility of the work that we publish. This form provides structure for consistency and transparency in reporting. For further information on Nature Research policies, see Authors & Referees and the Editorial Policy Checklist .

Statistics

For all statistical analyses, confirm that the following items are present in the figure legend, table legend, main text, or Methods section.

n/a	Confirmed	
☐	☒	The exact sample size (*n*) for each experimental group/condition, given as a discrete number and unit of measurement
☐	☒	A statement on whether measurements were taken from distinct samples or whether the same sample was measured repeatedly
☐	☒	The statistical test(s) used AND whether they are one- or two-sided<br>*Only common tests should be described solely by name; describe more complex techniques in the Methods section.*
☒	☐	A description of all covariates tested
☐	☒	A description of any assumptions or corrections, such as tests of normality and adjustment for multiple comparisons
☐	☒	A full description of the statistical parameters including central tendency (e.g. means) or other basic estimates (e.g. regression coefficient) AND variation (e.g. standard deviation) or associated estimates of uncertainty (e.g. confidence intervals)
☐	☒	For null hypothesis testing, the test statistic (e.g. *F*, *t*, *r*) with confidence intervals, effect sizes, degrees of freedom and *P* value noted<br>*Give P values as exact values whenever suitable.*
☒	☐	For Bayesian analysis, information on the choice of priors and Markov chain Monte Carlo settings
☒	☐	For hierarchical and complex designs, identification of the appropriate level for tests and full reporting of outcomes
☒	☐	Estimates of effect sizes (e.g. Cohen's *d*, Pearson's *r*), indicating how they were calculated

Our web collection on statistics for biologists contains articles on many of the points above.

Software and code

Policy information about availability of computer code

Data collection	No software was used for data collection.
Data analysis	Phoenix® WinNonlin® version 8.0, GraphPad Prism v.7.01, SAS version 9.4 software, STAR aligner v2.7.5, ShapeMapper v2.1.5

For manuscripts utilizing custom algorithms or software that are central to the research but not yet described in published literature, software must be made available to editors/reviewers. We strongly encourage code deposition in a community repository (e.g. GitHub). See the Nature Research guidelines for submitting code & software for further information.

Data

Policy information about availability of data

All manuscripts must include a data availability statement. This statement should provide the following information, where applicable:

- Accession codes, unique identifiers, or web links for publicly available datasets
- A list of figures that have associated raw data
- A description of any restrictions on data availability

GRCm38 mouse genome was retrieved from GENCODE (https://www.gencodegenes.org/mouse/).

Field-specific reporting

Please select the one below that is the best fit for your research. If you are not sure, read the appropriate sections before making your selection.

☒ Life sciences ☐ Behavioural & social sciences ☐ Ecological, evolutionary & environmental sciences

For a reference copy of the document with all sections, see nature.com/documents/nr-reporting-summary-flat.pdf

Life sciences study design

All studies must disclose on these points even when the disclosure is negative.

Sample size	Sample sizes were determined based on power calculations using a two-tailed unpaired t-test assuming equal variances between treatment vs. control groups to observe a >=50% or 75% decrease in primary disease plasma biomarkers assuming 80% power and a 5% significance level.<br>The study to characterize the kinetics of mRNA-encoded protein and enzyme were based on reasonable sample sizes per sacrifice time point consistent with the pharmacokinetics field. Basically, PA mice were sacrificed at each time point and for each study arm (dual mRNAs vs luc mRNA) and thus a large sample size was required (n=48 total) for this study. The purpose of this study was to characterize the kinetics of the mRNA-encoded protein and enzyme activity, and not to estimate precise population pharmacokinetic parameters. As the PK trends were consistent across all three evaluated parameters (2 protein subunits and enzyme activity) and also consistent with the kinetics of the pharmacodynamics data, we believe this sample size (n=3 for Luc and n=4 for dual mRNAs) was sufficient to characterize the kinetics for these mRNA-encoded proteins and enzyme activity.
Data exclusions	For the 6-month study data shown in Fig. 4d-e, data from n=5 instead of 6 WT mice were used due to exclusion of one outlier. No other data were excluded.
Replication	Key findings described in the Abstract were successfully replicated in a minimum of two separate experiments.
Randomization	The animals were randomly assigned to the groups based on age and weight. Where possible, randomization was also assigned based on gender.
Blinding	Where possible, all laboratory parameters (specifically primary disease biomarkers and clinical chemistry parameters) were quantified in a blinded fashion.

Reporting for specific materials, systems and methods

We require information from authors about some types of materials, experimental systems and methods used in many studies. Here, indicate whether each material, system or method listed is relevant to your study. If you are not sure if a list item applies to your research, read the appropriate section before selecting a response.

Materials & experimental systems

n/a	Involved in the study	
☐	☒	Antibodies
☐	☒	Eukaryotic cell lines
☒	☐	Palaeontology
☐	☒	Animals and other organisms
☒	☐	Human research participants
☒	☐	Clinical data

Methods

n/a	Involved in the study	
☒	☐	ChIP-seq
☒	☐	Flow cytometry
☒	☐	MRI-based neuroimaging

Antibodies

Antibodies used	For co-localization analysis, an anti-PCCA mouse monoclonal antibody (Santa Cruz #sc-393527), an anti-PCCB rabbit polyclonal antibody (Novus #NBP1-85886), and an anti-TOM20 mitochondrial marker antibody (Abcam #ab205486) were used. For capillary electrophoresis, a polyclonal anti-PCCA antibody (Proteintech #21988-1-AP), a polyclonal anti-PCCB antibody (Novus #NBP1-85886) and a monoclonal anti-β-actin antibody (Sigma #A2228-200UL) were used. For mitochondrial study via capillary electrophoresis, a polyclonal anti-CAT antibody (Abcam #ab52477), a polyclonal anti-MnSOD antibody (Enzo #ADI-SOD-110), a polyclonal anti-GPx1 antibody (Abcam #ab22604), a polyclonal anti-OGG1 antibody (Proteintech #15125-1-AP), a monoclonal anti-CypD antibody (Abcam #ab110324), and a monoclonal anti-GAPDH antibody (Abcam #ab8245) were used.
Validation	- anti-PCCA mouse monoclonal antibody (Santa Cruz #sc-393527): antibody was raised against amino acids 447-728 mapping at the C-terminus of PCCA of human origin. (https://www.scbt.com/p/pcca-antibody-h-1)<br>- polyclonal anti-PCCA antibody (Proteintech #21988-1-AP): Immunogen is PCCA fusion protein Ag17271 (https://www.ptglab.com/products/PCCA-Antibody-21988-1-AP.htm).<br>- anti-PCCB rabbit polyclonal antibody (Novus #NBP1-85886): This antibody was developed against Recombinant Protein corresponding to 80 amino acids at C-terminus (aa443 – 522) of human PCCB: SSKHLCGDTNYAWPTAEIAVMGAKGAVEIIFKGHENVEAAQAEYIEKFANPFPAAVRGFVDDIIQPSSTRARICCDLDVL (https://www.novusbio.com/products/pccb-antibody_nbp1-85886).<br>- anti-TOM20 antibody (Abcam #ab205486): Immunogen is recombinant fragment within Human TOMM20 aa 1 to the C-terminus (https://www.abcam.com/tomm20-antibody-epr15581-39-mitochondrial-marker-alexa-fluor-488-ab205486.html).<br>- monoclonal anti-β-actin antibody (Sigma #A2228-200UL): Immunogen is slightly modified β-cytoplasmic actin N-terminal peptide, Ac-Asp-Asp-Asp-Ile-Ala-Ala-Leu-Val-Ile-Asp-Asn-Gly-Ser-Gly-Lys, conjugated to KLH (https://www.sigmaaldrich.com/catalog/product/sigma/a2228?lang=en&region=US).<br>- polyclonal anti-CAT antibody (Abcam #ab52477): Immunogen is synthetic peptide corresponding to residues (aa 50-150) near

the N terminus of human Catalase. (https://www.abcam.com/catalase-antibody-peroxisome-marker-ab52477.html)
- polyclonal anti-MnSOD antibody (Enzo #ADI-SOD-110): Immunogen is human Mn SOD protein. (https://www.enzolifesciences.com/ADI-SOD-110/mn-sod-polyclonal-antibody/)
- polyclonal anti-GPx1 antibody (Abcam #ab22604): Immunogen is synthetic peptide within Human Glutathione Peroxidase 1 aa 150 to the C-terminus conjugated to keyhole limpet haemocyanin. The exact sequence is proprietary. (https://www.abcam.com/glutathione-peroxidase-1-antibody-ab22604.html)
- polyclonal anti-OGG1 antibody (Proteintech #15125-1-AP): Immunogen is OGG1 fusion protein Ag7204. (https://www.ptglab.com/products/OGG1-Antibody-15125-1-AP.htm)
- monoclonal anti-CypD antibody (Abcam #ab110324): Immunogen is recombinant full length protein corresponding to Rat Cyclophilin F aa 1-206. (also known as CypD). (https://www.abcam.com/cyclophilin-f-antibody-e11ae12bd4-ab110324.html)
- monoclonal anti-GAPDH antibody (Abcam #ab8245): Immunogen is rabbit muscle GAPDH. (https://www.abcam.com/gapdh-antibody-6c5-loading-control-ab8245.html)

Eukaryotic cell lines

Policy information about cell lines

Cell line source(s)

- Hep3B cell lines and normal human fibroblasts were from ATCC.
- Human fibroblasts, isolated from a PCCA-deficient PA patient (#GM371 cell line with PCCA mutations c.1788G>A [p.W596X] and c.1561-1566delinTATTGCCAATAACC) and a PCCB-deficient PA patient (#GM1298 cell line with PCCB mutations c.1218_1231delinsTAGAGCACAGGA and c.1606A>G [p.N536D]), were purchased from Coriell.

Authentication

None of the cell lines used here were authenticated.

Mycoplasma contamination

All cell lines were tested negative for mycoplasma contamination.

Commonly misidentified lines
(See ICLAC register)

No commonly misidentified cell lines were used in the study.

Animals and other organisms

Policy information about studies involving animals; ARRIVE guidelines recommended for reporting animal research

Laboratory animals

All the Pcca-/-(A138T) homozygous, Pcca+/- heterozygous and WT mice were on a FVB background strain. Mice used in all studies were mixed gender with the exception of a few studies where only female mice were used as they have elevated metabolite concentrations, i.e. plasma ammonia, compared to male mice. Mice were 4-5 months old at the initiation of all in vivo studies. Mice were housed under the following conditions: temperature - 68F to 79F (20C to 26C), humidity - 30% to 70%, dark/light cycle - an automatically controlled 12-hour light:12-hour dark light cycle was maintained.

Wild animals

The study did not involve the wild animals.

Field-collected samples

The study did not involved the samples collected from the field.

Ethics oversight

Experimental protocols were approved by the Institutional Animal Care and Use Committee at Moderna.

Note that full information on the approval of the study protocol must also be provided in the manuscript.
